# Highly hydrophilic and dispersed TiO_2_ nano-system with enhanced photocatalytic antibacterial activities and accelerated tissue regeneration under visible light

**DOI:** 10.1186/s12951-023-02241-2

**Published:** 2023-12-20

**Authors:** Boyao Lu, Jie Zhang, Guixin Zhu, Tiqian Liu, Jinwei Chen, Xing Liang

**Affiliations:** 1 State Key Laboratory of Oral Diseases & National Center for Stomatology & National Clinical Research Center for Oral Diseases, Department of Prosthodontics II of West China Hospital of Stomatology, Sichuan University, Chengdu, 610041 Sichuan China; 2 College of Materials Science and Engineering, Sichuan University, Chengdu, 610065 China; 3 Engineering Research Center of Alternative Energy Materials & Devices, Ministry of Education, Sichuan University, Chengdu, 610065 China

**Keywords:** Antimicrobial, Hydrophily, Wound healing, Photodynamic agents, Nanomaterial

## Abstract

Titanium dioxide (TiO_2_)-based photodynamic antibacterial (PDA) agents present a novel approach for addressing drug-resistant bacterial infections and the associated tissue damage. However, the suboptimal dispersibility, negative charge, and weak photocatalytic activity under visible light of TiO_2_ hinder its practical applications. This study aimed to address these limitations by developing a highly hydrophilic and dispersed Zn-TiO_2_/reduced graphene oxide (rGO) (HTGZ) nano-system with exceptional visible light catalytic activity and tissue repair ability. HTGZ produced an antibacterial ratio over 98% within a short time, likely due to the enhanced production of reactive oxygen species under visible light. After being co-cultured for 4 days, L929 cells and BMSCs maintained over 90% activity, indicating that HTGZ had no significant cytotoxicity. Furthermore, the transcriptomic and metabolic analyses revealed that the antibacterial mechanism mainly came from the destruction of cell membranes and the disruption of various metabolic processes, such as purine metabolism and fatty acid biosynthesis. Critically, results of in vivo experiments had authenticated that HTGZ significantly promoted infected tissue regeneration by slaughtering bacteria and release Zn^2+^. After 14 days, the wound area was only one-third that of the control group. Overall, the enhanced antibacterial efficacy and wound-healing potential position HTGZ as a promising nano-antibacterial medication for the clinical treatment of infectious bacterial diseases.

**Supplementary Information:**

The online version contains supplementary material available at 10.1186/s12951-023-02241-2.

## Introduction

Infectious bacterial diseases have long posed a serious threat to global healthcare [1, 2]. The gravity of this situation has been further exacerbated by antibiotic overuse, leading to the emergence of drug-resistant bacterial strains [3]. Projections indicate that the annual mortality rate attributable to antimicrobial resistance may escalate to a staggering 10 million by the year 2050 [4]. Consequently, there is an urgent and pressing need for substantial efforts aimed at the development of novel antibiotic-free strategies.

Recently, photodynamic antibacterial (PDA) agents based on photochemical reactions have garnered considerable attention [5]. Generally, photocatalysts are activated upon exposure to light to generate reactive oxygen species (ROS), which in turn induce oxidative damage to biomacromolecules that culminate in bacterial demise and markedly diminishes the likelihood of bacterial drug resistance [6, 7]. Furthermore, PDA agents exhibit advantages such as broad-spectrum bactericidal efficiency, shorter treatment durations and stronger bacterial targeting when compared to conventional antibiotic therapies [8]. Nevertheless, the biological toxicity of photodynamic materials cannot be ignored, as ROS can also harm normal tissue cells. Element doping and material size can both affect the magnitude of toxicity, which also hinders the practical application of these materials [9, 10].

Titanium dioxide (TiO_2_) is a commonly used photocatalyst in solar cells, photocatalytic water splitting, and the degradation of organic pollutants [11]. Owing to its stable chemical properties and good biocompatibility, it has attracted widespread attention in biomedical research [12]. However, due to the wide bandgap, low carrier separation, and high recombination rates, the ROS production of TiO_2_ under visible light is very limited [13]. In order to achieve significant antibacterial effects, higher concentrations or longer exposure times are needed; however, these can significantly increase the biological toxicity of TiO_2_, leading to altered protein function and cell activity [14, 15]. Because PDA-produced ROS have a limited diffusion distance, materials need to adhere to the bacterial surface to exert effects [16]. As a result, it is necessary to account for the dispersibility of PDA materials in a matrix in biomedical applications. The surface of bacterial cells is negatively charged [17]. A positive charge of materials is also important for strong electrostatic interactions with bacteria.

The modification of TiO_2_ with carbon holds considerable promise owing to the great chemical stability, cost-effectiveness, and electronic acceptor properties of carbonaceous materials [18]. When exposed to light, the modified material can transfer electrons to the conduction band, thereby broadening the absorption range to the visible light region and improving the light absorption capacity [19]. The carbon compounds on the TiO_2_ surface can also enhance the hydrophilicity of materials. Doping with metal ions is a common modification that can adjust the energy-level structure of TiO_2_, thus enhancing carrier separation efficiency [20, 21]. Among these dopants, Zn^2+^ exhibits favorable effects on epithelial formation and therefore can improve the TiO_2_-based PDA applications to infected wound healing [22]. Graphene and its derivatives are widely used in various biomedical fields [23]. Two-dimensional (2D) graphene can endow TiO_2_ with superior electron mobility, which promotes carrier separation and ROS output [24]. In addition, rGO-related antibacterial materials have attracted research attention due to the sharp edge-mediated cutting and oxidative stress effects of rGO [25].

**Scheme 1:**
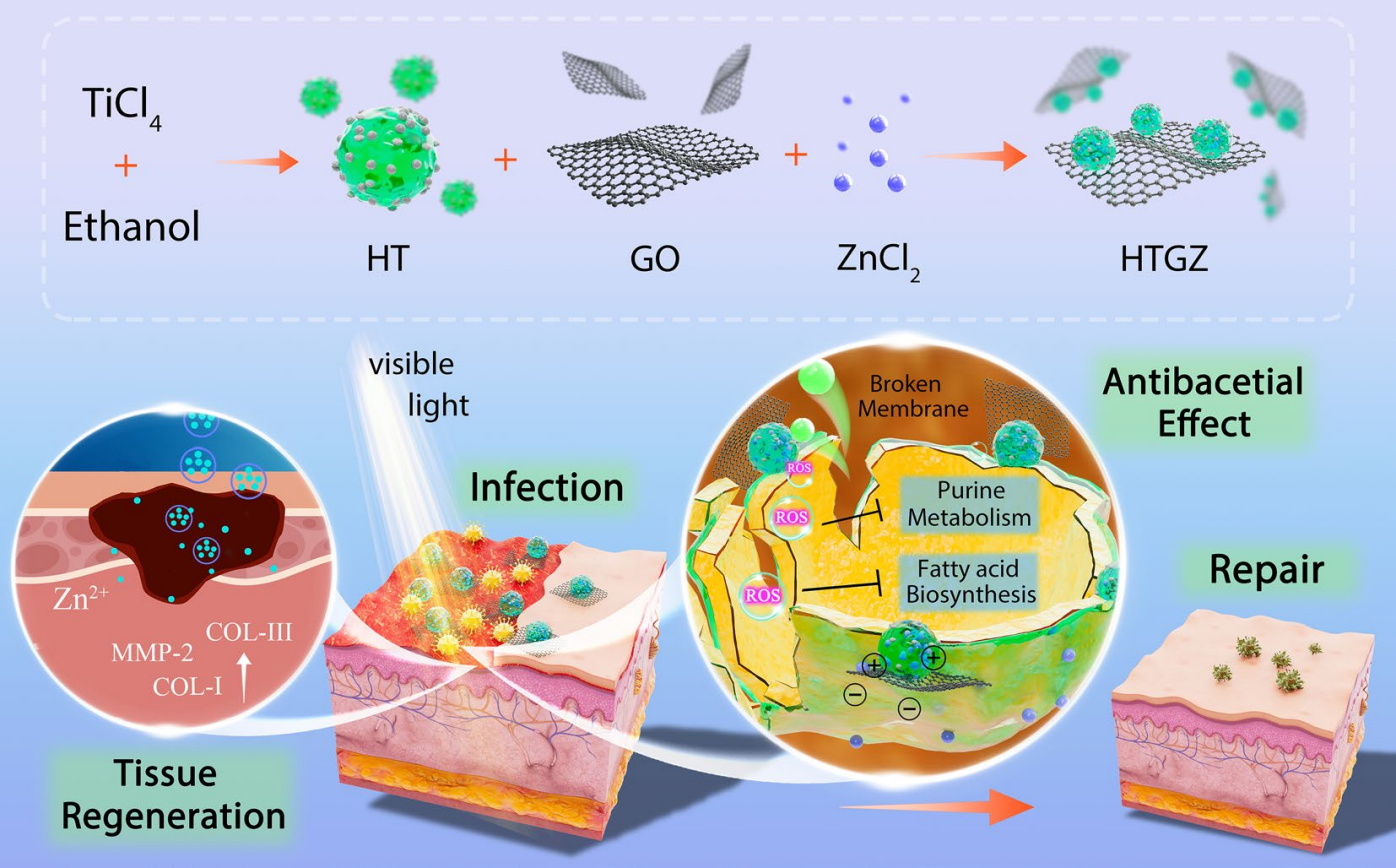
Schematic diagram of HTGZ nano-system with enhanced photodynamic antibacterial effect and accelerated infected wound repair ability

In this study, we developed novel carbon-modified TiO_2_ nanoparticles with excellent hydrophilicity, dispersibility, positive charges, and visible light responses (denoted as HT). HT was uniformly dispersed on the reduced graphene oxide (rGO) to achieve an HT/rGO (HTG) species with improved photodynamic performance. Moreover, the preparation process involved simultaneous doping of Zn^2+^ into HT to obtain a Zn-HT/rGO (HTGZ) nano-system with improved biological effects. We speculate that the hydrophilicity, dispersibility, and positive charge of HTGZ facilitated improved diffusion and contact with bacterial cells for maximal effectiveness. Increased ROS production under visible light conditions will enable the material to exhibit stronger antibacterial effects. In addition, due to the action of zinc ions, the material can effectively promote the healing of infected wounds with less cytotoxicity. As expected, this design and combination improved the properties of PDA under visible light and the injury repair activities of the nanomaterial, and satisfactory results were achieved in both in vivo and in vitro experiments. Furthermore, we also explored the mechanisms underlying its antibacterial effects via transcriptomic and untargeted metabolic analyses. In general, HTGZ has tremendous research value as a promising antibacterial material (Scheme 1).

## Materials and methods

Materials and methods are presented in Additional file 1.

## Results and discussion

### Morphology and structure characterization

**Fig. 1:**
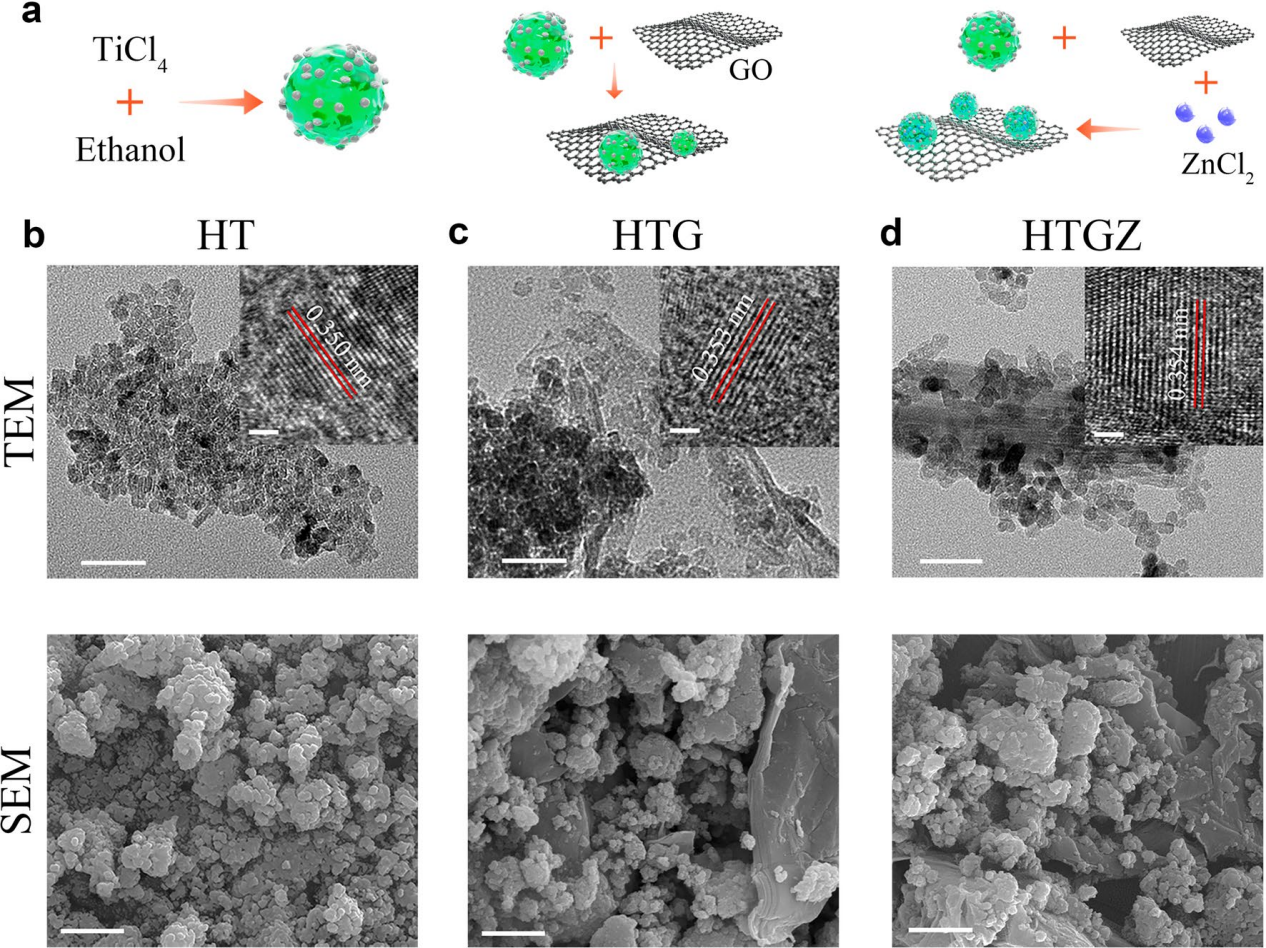
Fabrication and morphology of nanomaterials. **a** Schematic illustration of the preparation procedure. **b**–**d** TEM, HR-TEM and SEM images of nanomaterials. Scale bars are 50 nm for TEM, 2 nm for HR-TEM and1 µm for SEM

Figure 1a schematically illustrates the preparation procedure of the HTGZ nano-system. HT was initially prepared from TiCl_4_ and ethanol using a nonaqueous sol–gel method. Subsequently, graphene oxide and ZnCl_2_ were added into HT to produce HTG and HTGZ, respectively.

**Fig. 2:**
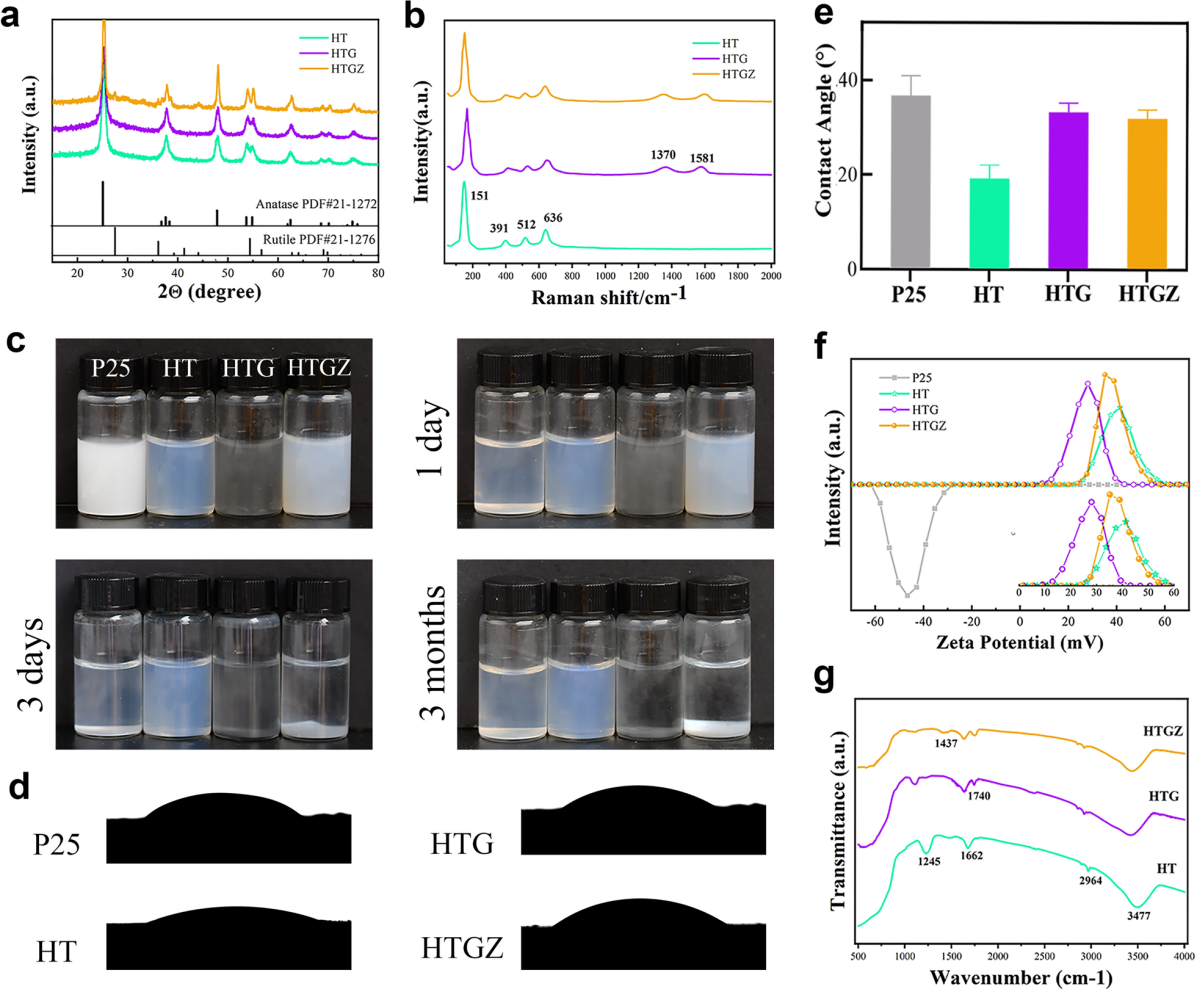
Characterization of nanomaterials. **a** XRD patterns of nanomaterials. **b** Raman spectra of nanomaterials. **c** Photographs of nanomaterials with concentrations of 1% (w/v) left to rest for 1 day, 3 days and 3 months, respectively. **d**, **e** Contact angle test for nanomaterials. The data are presented as mean ± SD (n = 3). **f** The zeta potential of nanomaterials. **g** FTIR spectra of nanomaterials

Scanning electron microscopy (SEM) images revealed that HT was composed of agglomerated particles (Fig. 1b). Transmission electron microscopy (TEM) images showed compact nanoparticles with the size of approximately 10 nm. A lattice spacing of 0.350 nm was observed using high-resolution TEM (HR-TEM), which corresponded to the (101) crystal plane of anatase TiO_2_. TEM image of HTG showed some particles embedded in the wrinkled thin-layers, demonstrating the successful combination of rGO (Fig. 1c). Energy dispersive spectroscopy (EDS)-mapping images further manifested the homogeneous distributions of C, N, O, and Ti elements across the whole HTG (Additional file 1: Fig. S1). The morphology of HTGZ was similar to that of HTG (Fig. 1d), and the elemental mapping confirmed the successfully Zn doping with the composition ratio of 2.6 wt% (Additional file 1: Fig. S2). HR-TEM analysis of HTGZ revealed a lattice fringe with a calculated spacing of 0.354 nm, indicating a distinct lattice distortion of TiO_2_ attributed to the introduction of Zn [26]. Furthermore, X-ray diffraction (XRD) patterns of HT displayed characteristic diffraction peaks of anatase TiO_2_ (Fig. 2a), which remained unaffected in terms of position and intensities of HTG and HTGZ, suggesting that the crystal structure of TiO_2_ was unaltered by the presence of rGO and Zn. Raman spectroscopy was employed to examine the vibration modes of rGO, revealing characteristic peaks at approximately 151, 391, 512, and 636 cm^−1^ that were consistent with TiO_2_ features. The peaks at 1370 cm^−1^ and 1581 cm^-1^ represented the rGO in HTG (Fig. 2b). These results preliminarily indicated that HTGZ had been successfully synthesized.

As shown in Fig. 2c. The P25 (commercial TiO_2_) nanoparticle, at a suspension concentration of 0.1% (w/v), precipitated completely after 1 d of rest without any stirring or disturbance, whereas suspensions of HTG and HTGZ remained stable for approximately 3 d. Notably, HT exhibited long-term high dispersibility for up to 3 months. Unevenly dispersed particles caused by sedimentation decrease the biological function of nano-systems dramatically; therefore, the high dispersibility of the nano-system is favorable for biological applications. Contact angles were determined to evaluate the hydrophilicity of the nanomaterials (Fig. 2d, e). HT had a smaller water contact angle (31.5°) than that of P25 (37.5°). However, the contact angles of HTG and HTGZ increased to 36.1° and 34.8°, respectively, which can be ascribed to the addition of hydrophobic graphene. Unlike P25 (− 46.4 mV), HT, HTG, and HTGZ exhibited positive zeta potentials of 28.6, 41.2, and 35.7 mV, respectively (Fig. 2f). The positive charge of nanomaterials facilitates adherence to bacterial surfaces with a negative charge. A high positive zeta potential also reduces aggregation and maintains physical stability [27]. Based on these findings, the combination of the favorable hydrophilicity, dispersibility, and positive potential synergistically promote the complete and tight interaction between HTGZ and cells, potentially leading to enhanced antibacterial effects and the reliable evaluation of its biological activity.

Fourier transform infrared spectroscopy (FTIR) and X-ray photoelectron spectroscopy (XPS) were further used to explore the chemical compositions of the nanomaterials. The FTIR spectrum of HT revealed C-H stretching vibrations at 2926 cm^−1^ attributed to the hydrocarbon moiety. Peaks at 1715 cm^−1^ and 1104 cm^−1^ corresponded to C=O in oxygenated functional groups and C–O groups, respectively (Fig. 2g). These results suggested that carbon oxide compounds were generated in HT during the carbonization of ethanol [28]. The peaks at approximately 1635 cm^−1^ in HTG and 1652 cm^−1^ in HTGZ corresponded to the stretching vibrations of C=C in rGO. XPS survey spectra (Additional file 1: Fig. S3a) indicated similar peaks for C, Ti, and O elements in HTG and HTGZ. The Ti 2p peaks in HT could be divided into Ti 2p_1/2_ (464.3 eV) and Ti 2p_3/2_ (458.6 eV) of Ti^4+^ in TiO_2_ (Additional file 1: Fig. S3b). The dominant peak at 529.8 eV in the O 1s was related to the lattice oxygen in TiO_2_, and the other peak suggested the presence of other oxygenated compounds (Additional file 1: Fig. S3c) [29]. Curve fitting of the C 1s spectra resulted in three peaks at 284.4, 285.6, and 288.9 eV (Additional file 1: Fig. S3d), where the peaks at 285.6 and 288.9 eV were assigned to the carbon bound to oxygen (C–O and C=O), and the additional peak possibly originated from carbon on the nanoparticle surface.. These results were consistent with the FTIR results, confirming that carbonaceous species and oxygenated functional groups existed at the surface of HT, which resulted in a high surface potential, prevented nanocrystal agglomeration, and ultimately led to excellent hydrophilicity and dispersibility. In the HTG spectrum, the C=C bond in the rGO may have contributed to the significantly elevated peak at 284.7 eV (Additional file 1: Fig. S3e). Regarding HTGZ, two peaks with binding energies of 1022.2 and 1045.2 eV represented Zn 2p_1/2_ and Zn 2p_3/2_, demonstrated that Zn was bivalent (Additional file 1: Fig. S3f). Results of chemical composition analysis for nanomaterials further demonstrated that the HTGZ nano-system had been successfully synthesized as we designed.

### Photodynamic properties and mechanism

**Fig. 3:**
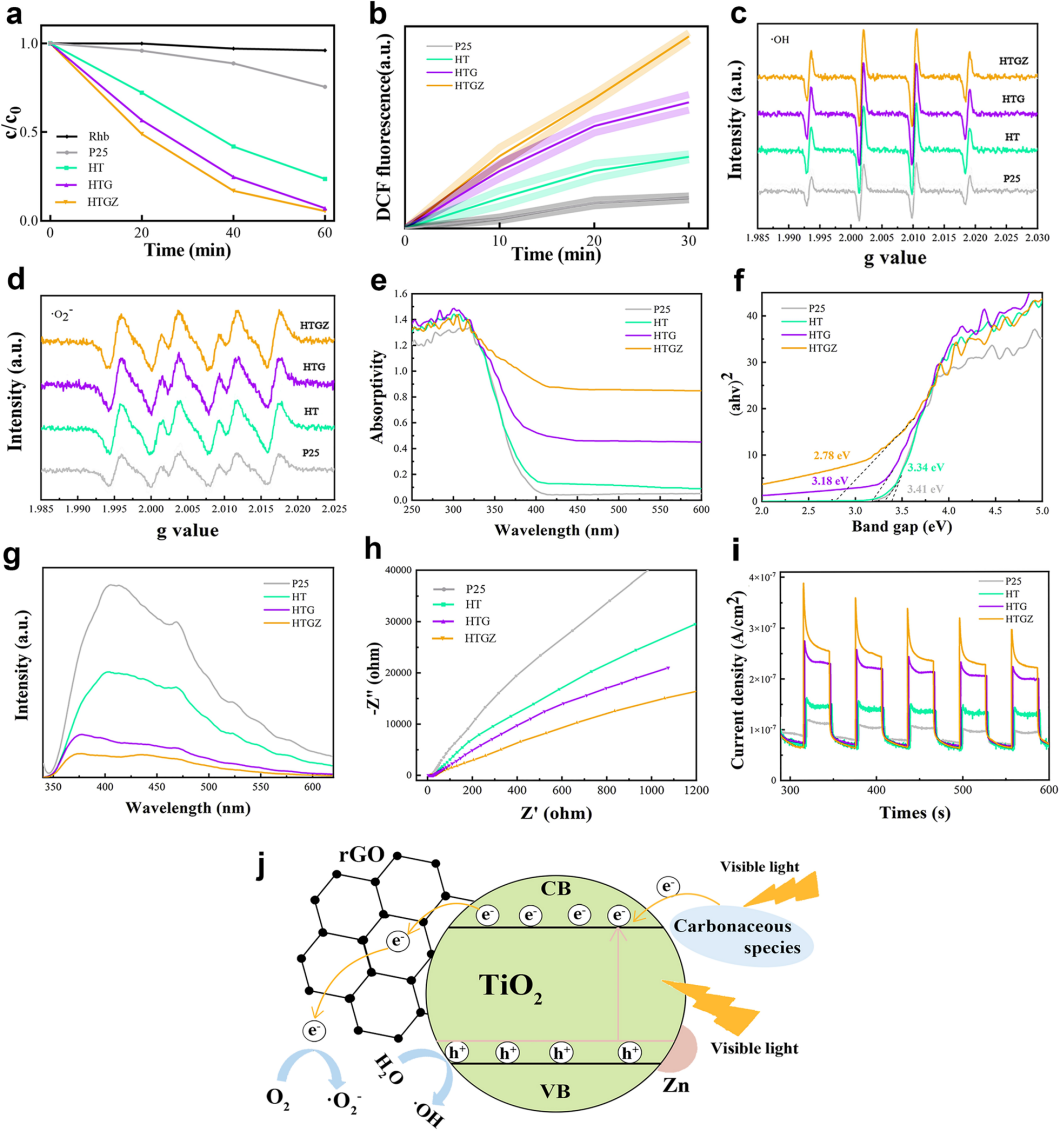
Photodynamic properties of nanomaterials. **a** Absorbance of RhB self-degradation in the absence of light, as well as under visible light irradiation for nanomaterials. **b** ROS production of nanomaterials under visible light irradiation. **c**, **d** ESR for ·OH and ·O^2−^ generation of nanomaterials. **e** UV–vis–NIR absorption curves of nanomaterials. **f** Bandgap of nanomaterials. **g** PL spectra of nanomaterials. **h** Electrochemical impedance values of nanomaterials under visible light irradiation. **i** Photocurrent curves of nanomaterials. **j** Proposed schematic diagram of HTGZ for ROS production

To evaluate the PDA activity of the nanomaterials, the corresponding photodynamic performance were systematically evaluated. The HTGZ exhibited the best degradation efficiency on RhB under visible light (Fig. 3a), indicating its superior photocatalytic activity. Similarly, as shown in Fig. 3b, the higher fluorescence intensity of the HTGZ indicated better ROS production than those of P25 and other contrast samples. Furthermore, electron spin resonance spectra revealed that ·OH and ·O^2−^ production in the HTGZ was greatly enhanced when compared with other contrasts (Fig. 3c, d). The results provide direct evidence that HTGZ exhibits an outstanding photodynamic performance under visible light.

To elucidate the mechanism of the enhanced photodynamic performance, UV–vis–near-infrared and photoluminescence (PL) spectra were performed. The light absorption of P25 was poor in the range 300–700 nm, and the enhancement of HT was also limited. However, a redshift in the absorption edge occurred and a higher absorption rate was clearly observed for HTG and HTGZ (Fig. 3e). Correspondingly, the band gap gradually reduced from 3.41 to 2.78 eV, leading to better absorption efficiency in the visible light range (Fig. 3f). The previous studies indicated that a significant reduction of band gap may improve the photogeneration of electron–hole pair [30]. Therefore, the HTGZ facilitates the enhancement of the light absorption capacity and the separation efficiency of electron-hole pairs, thus may result in enhanced photocatalytic performance. PL spectra can be used to detect the separation efficiency of photogenerated carriers based on the fluorescence intensity. Stronger fluorescence indicates that electrons and holes are more likely to be compounded [31]. As shown in Fig. 3g, the as-prepared TiO_2_-based nanomaterials exhibit weaker fluorescence and reduced recombination efficiency compared to P25. Later, photocurrent and electrochemical impedance measurements were performed to probe the electron-hole pair separation ability. The Nyquist plot from electrochemical impedance spectroscopy (Fig. 3h) reveals that the HTGZ possess the lowest resistance owing to the smaller circle diameter than those of contrast samples, indicating the fastest charge transfer efficiency under visible light irradiation. Furthermore, the enhanced photocurrent density of HTGZ (Fig. 3i) was much higher than those of contrast samples, demonstrates an increased photogenerated carrier separation [32]. In general, the accelerated electron transfer and abundant oxygen supply are considered as crucial factors to improve photodynamic performance [33]. The above results confirm that the HTGZ exhibits better ROS production, fast electron transfer, and a decreased energy barrier, thus leading to a faster photodynamic reaction.

The enhanced photodynamic mechanism of HTGZ is illustrated in Fig. 3j. On the one hand, the electrons transferred to the conduction band of TiO_2_ through the sensitization of the carbonaceous species on the surface of HTGZ under visible light irradiation. The doping of Zn^2+^ introduces an impurity energy level and more defects. A narrower bandgap could increase the optical absorption to produce more charge carriers. On the other hand, rGO can act as an acceptor in electron transfer that receiving photogenerated electrons on TiO_2_, thereby achieving more efficient separation of photogenerated electron–hole pairs and restraining their recombination. These factors synergistically boost the ROS yields and promote the photodynamic capability of HTGZ under visible light.

### Photocatalytic antibacterial activity in vitro

**Fig. 4:**
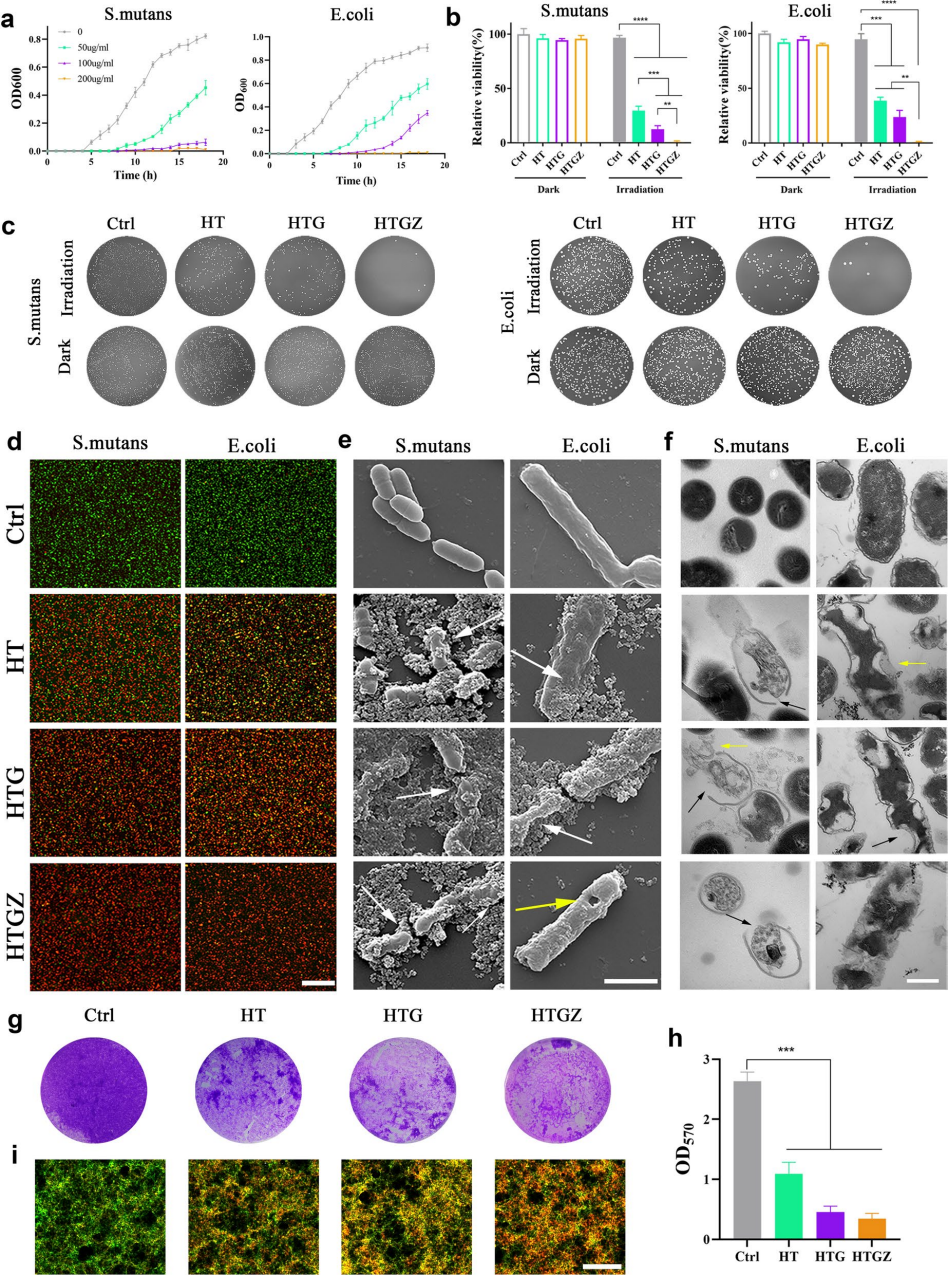
In vitro antibacterial effects of nanomaterials toward laboratory bacteria strains of *S. mutans* and *E. coli*. **a** The growth curve of bacteria after treatment with 200 µg mL^−1^ nanomaterials for 15 min. **b** Antibacterial ratios of 200 µg mL^−1^ nanomaterials against bacteria for 15 min. **c** Plate photographs of 200 µg mL^−1^ nanomaterials against bacteria for 15 min. **d** Live/dead staining images of bacteria after treatment with 200 µg mL^−1^ nanomaterials under 15 min of irradiation. Scale bars are 20 μm. **e** SEM images of bacteria after treatment with 200 µg mL^−1^ nanomaterials under 15 min of irradiation. Scale bars are 1 μm. **f** TEM images of bacteria after treatment with 200 µg mL^−1^ nanomaterials under 15 min of irradiation. Scale bars are 300 μm. **g**, **h** Photographs of crystal-violet-stained *S. mutans* biofilms with different treatments and its quantitative analysis. **i** Live/dead staining images of *S. mutans* biofilms after treatment with 200 µg mL^−1^ nanomaterials under 15 min of irradiation. Scale bars are 20 μm. The data are presented as mean ± SD (n = 3). Statistical significance: *P < 0.05; **P < 0.01; ***P < 0.001 and ****P < 0.0001

Gram-positive cocci (*Streptococcus mutans*) and gram-negative coliform bacteria (*Escherichia coli*) were used to determine antibacterial activities. First, the bacterial growth curve at an optical density (OD) of 600 nm was measured after visible light irradiation for 15 min (Fig. 4a, Additional file 1: Fig. S4a). The results showed that the nanomaterials had a significant inhibitory effect on both types of bacteria. The relative bacterial viability was detected on the colony-forming unit (CFU) counts. 200 µg mL^−1^ HT effectively killed *S. mutans* at an antibacterial rate of 70.26%. Owing to the increased production of ROS, the rate of HTG and HTGZ reached 87.64% and 98.25%, respectively. For *E. coli*, the antibacterial rate was 61.23%, 78.17%, and 99.15% for HT, HTG, and HTGZ, respectively (Fig. 4b). The photographs of bacterial colonies were shown in Fig. 4c. In addition, the loss of bacterial viability occurred in a concentration- and time-dependent manner (Additional file 1: Fig. S4b). Interestingly, the HTGZ exhibited stronger antibacterial effects against *S. mutans* than against *E. coli*. This is probably because gram-negative bacteria with dense outer membranes comprising lipopolysaccharides and lipoproteins show strong PDA resistance [34].

The live/dead bacterial fluorescence staining assay results were in accordance with the CFU assays. Bright green fluorescence indicated bacteria with intact membranes in the control group, while red fluorescence revealed damaged cells in the treatment groups (Fig. 4d).

SEM and TEM images were used to visualize the effect of nanomaterials on the morphological changes in bacteria. SEM images reveal that bacteria after treatment exhibited apparent wrinkling of the cell walls (white arrows), broken shape, and visible perforations (yellow arrow) In addition, the nanomaterials tightly adhered to the bacteria because of their hydrophilicity, which ensured that their PDA capacity was effective (Fig. 4e). TEM images clearly display the bacteria in the treated group showed distorted or broken membranes/cell walls (black arrows), and the intracellular material dispersed with outflow of the cytoplasm (yellow arrow) (Fig. 4f). It can be speculated that the PDA properties of nanomaterials are likely attributed to the direct damage to the bacterial structures.

The levels of adenosine triphosphate (ATP), as an essential energy molecule for microorganisms, decreased significantly, suggesting a stronger inhibitory effect on the viability of bacteria (Additional file 1: Fig. S5a). In addition, when the bacterial membrane/walls were broken, intracellular protein leaked from cells. The increase of extracellular protein concentration indicating that structural damages (Additional file 1: Fig. S5b).

In some cases, bacteria form an ordered and defensive biofilm that can prevent antibiotic penetration, decreasing the bactericidal ability of the antibiotics [35]. Thus, the ability of nanomaterials to inhibit *S. mutans* biofilm formation was investigated using crystal violet staining. In the treatment group, the staining of the biofilm was incomplete and light purple, indicating that biofilm formation was significantly inhibited; this was quantitatively confirmed by the OD_570_ value (Fig. 4g, h). Moreover, live/dead assays were used to detect the ability of nanomaterials to disrupt mature biofilms. The increase in red fluorescence in the treatment groups indicated that the nanomaterials could effectively eliminate biofilms, which is also consistent with the CFU results (Fig. 4i).

These results indicate that nanomaterials have good antibacterial properties, possibly due to the ability of ROS to disrupt bacterial membranes/cell walls and structures while also interfering with metabolic activities. In addition, rGO not only increases ROS production, but also has certain antibacterial properties. Sharp edge-mediated cutting and mechanical wrapping can damage the bacteria, which also contributes to the excellent antibacterial ability of HTGZ [36].

Due to the excellent antibacterial potential of GO, there have been many reports on GO-TiO_2_ photodynamic antibacterial composites [37, 38]. The combination of carbon and Zn^2+^ doping allow HTGZ to have excellent antibacterial activity even under low power irradiation (300 mWcm^−2^) and short action times (15 min). In addition, due to the antibacterial ability of Ag^+^, GO–TiO_2_–Ag composites have improved PDA effects [39]. Guo reported that 100 mg L^−1^ of this material could reduce *E. coli* survival to 12.2% in 10 min [40]. In contrast, HTGZ has lower biological toxicity and the ability to promote wound healing due to the function of Zn^2+^, thus having better biological application prospects.

### Exploration of antibacterial mechanism

**Fig. 5:**
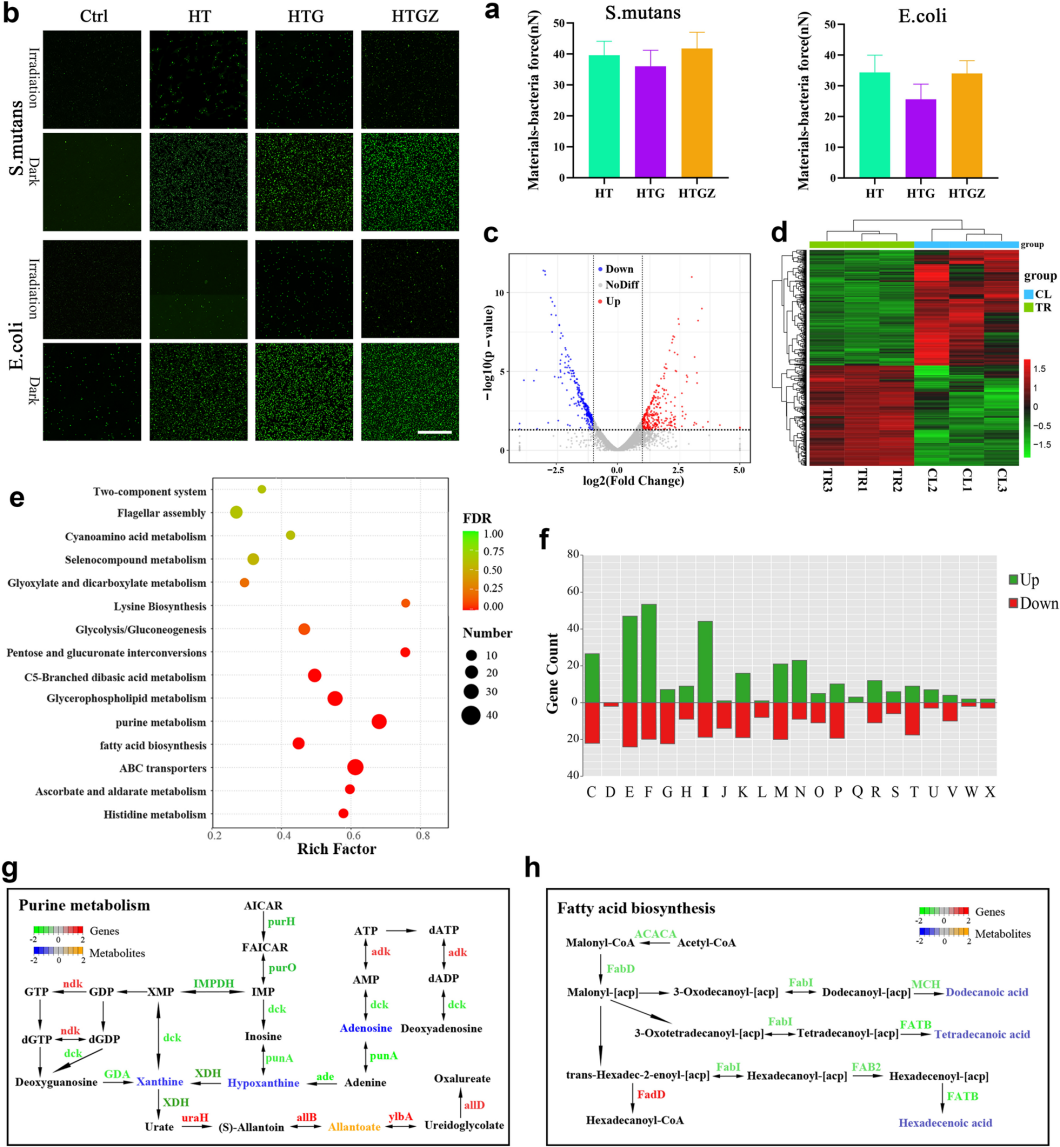
Exploration of antibacterial mechanisms of nanomaterials based on PDA. **a** Quantitative analysis of the interaction force between nanomaterials and bacterial cells examined by AFM. The data is presented as mean ± SD (n = 3). **b** ROS observation of bacteria incubated with nanomaterials with or without visible light irradiation. Scale bars are 50 μm. **c** Volcano plots identified the DEGs for CL vs. TR. **d** Heatmap of the DEGs. **e** KEGG enrichment for the DEGs of CL versus TR. **f** DEGs in CL versus TR by the COG functional categories. **g**, **h** Integrated purine metabolism and fatty acid biosynthesis pathways analysis of metabolome and transcriptome data. Gene expression with significant differences (P < 0.05) and distinct metabolic profiles were quantified as log2Foldchange

First, we examined the interaction force between bacteria and materials by atomic force microscopy (AFM) [41]. The results showed that the force was above 25 nN for *E. coli*, and reached about 40 nN for *S. mutans* (Fig. 5a, Additional file 1: Fig. S6). The nanomaterials with positive charge can easily achieve close integration with the negatively charged bacterial cells, thus ultimately enhancing bacterial inactivation efficiency.

We speculate that the antimicrobial effect of the nanomaterials was mainly due to the large amount of ROS. To verify this idea, the DCFH-DA probe was used to detect intracellular ROS levels [5]. As depicted in Fig. 5b, enhanced fluorescence intensity was observed in nanomaterial-incubated bacterial cells under illumination.

Accumulated intracellular ROS can overwhelm the bacterial antioxidant defense system and induce severe damage to various cellular substances crucial for normal cellular physiological activities. To further evaluate the changes in bacteria during this process, we conducted transcriptomic and untargeted metabolic analyses of *E. coli*. Principal component analysis (PCA) showed that gene expression in the HTGZ-treated (TR) group was well distinguished from that in the control (CL) group (Additional file 1: Fig. S7a). In total, 566 differentially expressed genes (DEGs) were identified as shown in the volcano plot and heat map (Fig. 5c, d). The Kyoto Encyclopedia of Genes and Genomes (KEGG) functional enrichment analysis revealed that purine metabolism, glycerophospholipid metabolism, glycolysis, amino acid metabolism and ATP-binding cassette (ABC) transporter family pathways were significantly affected (Fig. 5e). According to the clusters of orthologous groups (COGs) analysis, the genes involved in energy production and conversion (C), amino acid transport and metabolism (E), nucleotide transport and metabolism (F), and lipid transport and metabolism (I) were significantly changed (Fig. 5f). Branched-chain amino acids are necessary for the synthesis of membrane fatty acids [42], therefore, changes in lipid metabolism may result from the disruption of the cell membrane by ROS. As a transport ATPase on the cell membrane, ABC transporters were also affected [43]. In addition, excessive ROS disturbed purine metabolism and glycometabolism and also significantly reduced the production of ATP, which was also consistent with the results shown in Additional file 1: Fig. S5a.

Orthogonal partial least squares discriminant analysis (OPLS-DA) demonstrated a clear distinction between the metabolites in the TR and CL groups (Additional file 1: Fig. S7b). Among the differently expressed metabolites (DEMs), lipids and lipid-like molecules was the dominant class (Additional file 1: Fig. S7c). These results suggested that the synthesis of lipids and nucleosides was significantly affected by PDA. Subsequently, the combined KEGG pathway analysis of transcriptomics and metabolomics showed that the purine metabolism and fatty acid biosynthesis pathways were enriched with DEGs and DEMs (Fig. 5g, h). Most gene expression changes in these two pathways result in the decreased expression of several metabolites, such as hypoxanthine, adenine, and dodecanoic acid, ultimately leading to metabolic disorders and cell damage.

Based on these results, the potential PDA mechanism of nanomaterials under visible light can be summarized as follows: Due to their excellent hydrophilicity, dispersibility, and positive potential, nanomaterials can effectively accumulate on bacterial surfaces. Subsequently, excessive ROS beyond the cellular elimination capacity leads to the disruption of cell membranes and release of cellular contents. Meanwhile, normal metabolic processes, especially of purine metabolism and fatty acid biosynthesis pathways of bacteria is severely disrupted, ultimately leading to bacterial death.

### Cytotoxicity and cell migration ability assessment in vitro

**Fig. 6:**
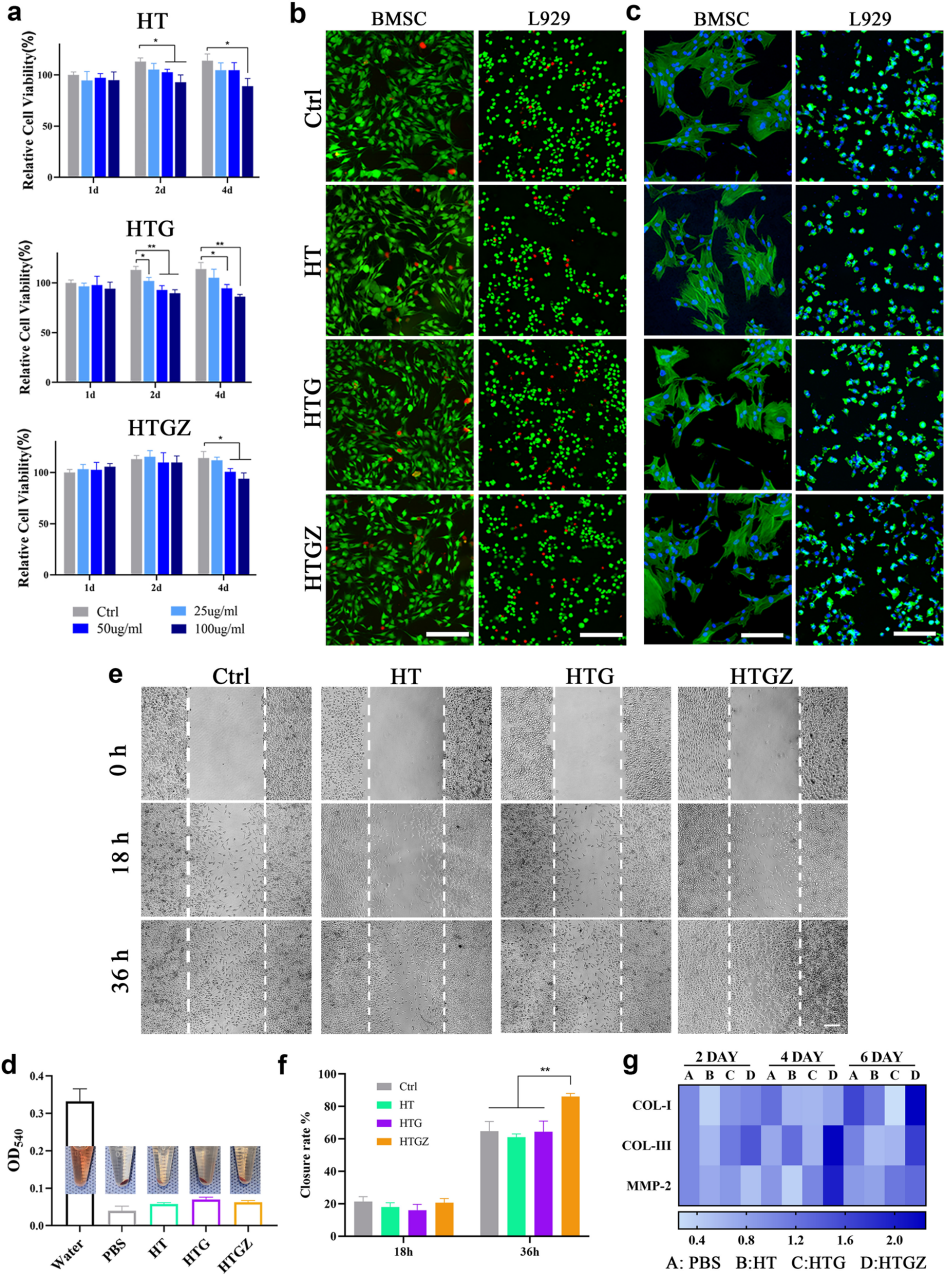
In vitro cytotoxicity and wound healing-related gene expressions. **a** CCK-8 results of L929 cells co-cultured with nanomaterials. **b** Live/dead staining images of cells co-cultured with nanomaterials for one day. Scale bars are 500 μm. **c** Morphology of cells co-cultured with nanomaterials for one day. Scale bars are 100 μm for BMSCs and 200 μm for L929 cells. **d** Hemolytic tests data and photographs of nanomaterials with rat blood cells. Scale bars are 100 μm. **e**, **f** Cell scratch assay of L929 cells images and the statistical analysis of closed area percentage. **g** Heat map of gene expression in L929 cells co-cultured with nanomaterials. The expression of PBS groups are the control groups. The data are presented as mean ± SD (n = 3). Statistical significance: *P < 0.05; **P < 0.01; ***P < 0.001 and ****P < 0.0001

L929 cells and rat bone marrow stem cells were used to assess cytotoxicity. HT and HTG showed no obvious cell growth inhibition after 1 d but exhibited some toxicity at 2 and 4 d when exposed to 50 or 100 µg mL^−1^. HTGZ promoted cell proliferation at lower concentrations, and the inhibitory effect of high concentrations on cell growth was weaker than that of HT or HTG (Fig. 6a, Additional file 1: Fig. S8). This enhancement may be ascribed to Zn^2+^ promotes cell growth and suppresses apoptosis. The live/dead cell fluorescence staining assay showed similar results (Fig. 6b). The morphology showed that cells in the treated groups spread well with a number of lamellipodia, similar to the control group. (Fig. 6c). These results indicate that the nanomaterial had no significant cytotoxicity.

Hemolytic test was performed to assess blood biosafety. The absorbance of the solution in the treated groups was similar to that of the negative control group (Fig. 6d), demonstrating negligible damage to the erythrocytes and good blood compatibility of the nanomaterials.

Scratch experiments can visually demonstrate the ability of cells to proliferate and migrate, which is crucial for wound healing. Due to the release of Zn^2+^, the migration and proliferation ability of cells in the HTGZ group was significantly improved when compared to the HT, HTG, and control groups (Fig. 6e, f).

To further investigate the mechanism of HTGZ in promoting wound healing, we conducted qRT-PCR to assess the expression of several tissue repair genes including matrix metalloproteinase-2 (*MMP-2*) and type I (*COL-I*) and type III (*COL-III*) collagen. After co-cultured with HTGZ for 4 d, the expression of COL-III increased by nearly two times compared to the other groups. After 6 d, *COL-I* and *MMP-2* expression also significantly increased (Fig. 6g). MMP-2 is a kind of polypeptides depending on Zn^2+^, which is important to the activation/migration of fibroblast over the extracellular matrix [44]. Collagen protein is the main constituent of skin connective tissue, so the high expression of COL-I and COL-III is important for wound repair [45]. This upregulated gene expression may explain why Zn^2+^ stimulates fibroblast movement. In addition, due to their higher surface energy, hydrophilic surfaces can enhance interactions between cells and proteins in the early stages of tissue regeneration [16]. The two-dimensional porous structure of rGO can serve as a scaffold for cell migration and adhesion [46]. These all contribute to how HTGZ treatment promotes cell migration.

During the wound healing process, the faster cell migration and improved collagen deposition induced by HTGZ can accelerate tissue regeneration, and the biosafety and biocompatibility of HTGZ make it a promising versatile platform for future biomedical anti-infective applications.

### Infected wound healing efficacy and toxicity in vivo

**Fig. 7:**
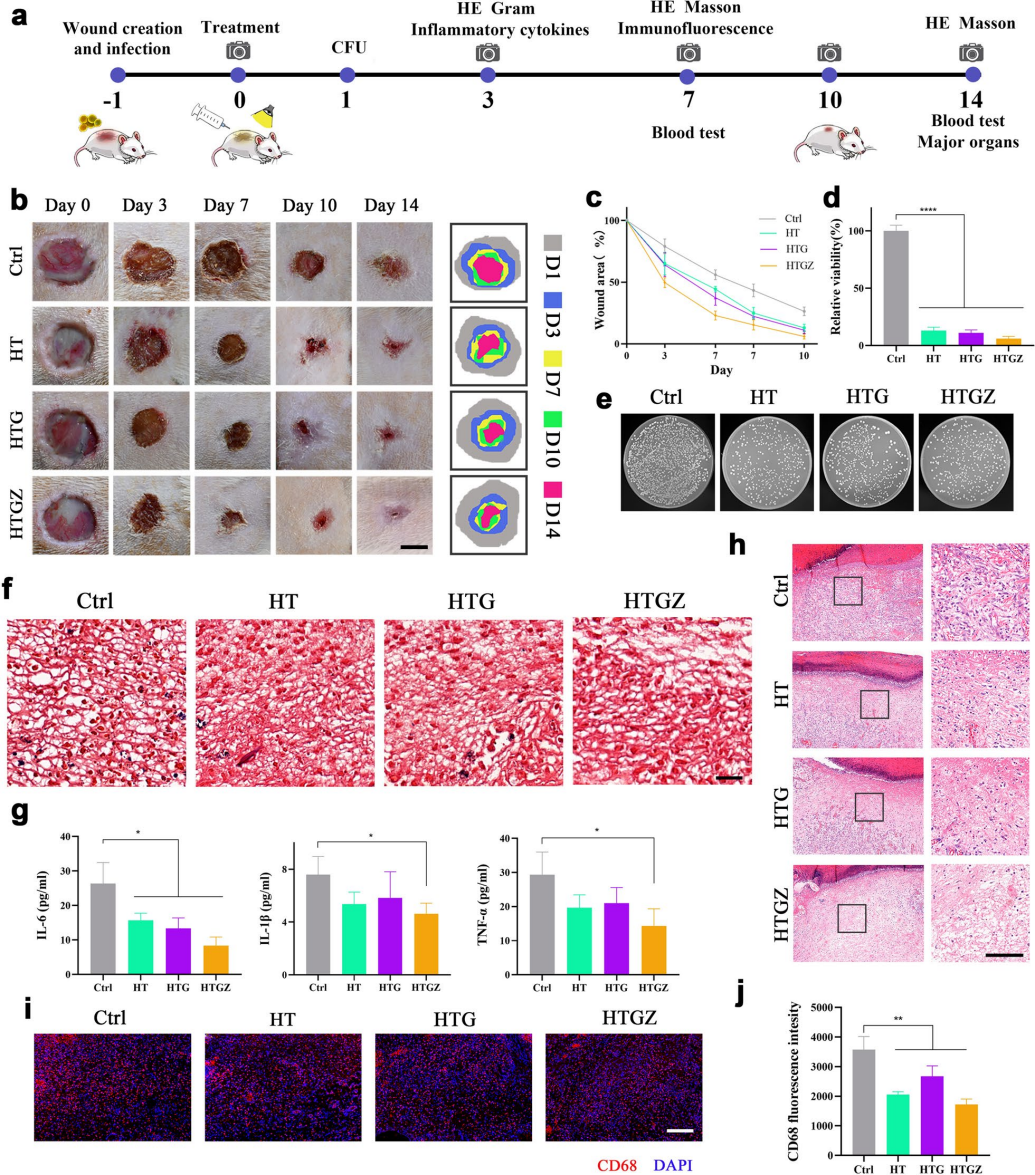
Antibacterial activity of nanomaterials in the infected wound model in vivo. **a** Schematic illustration showing the process of experiments about wound repair evaluation. **b** Representative photographs and schematic presentation of wound healing sites. Scale bars are 5 mm. **c** Quantitative analysis of wound area. **d**, **e** Quantitative analysis of CFUs and photographs of bacterial colonies of the infected wounds on day 1. **f** Gram staining of infected skin tissues on day 3. Scale bars are 50 μm. **g** Serum levels of IL-1b, IL-6, and TNF-α in blood samples collected on day 3. **h** H&E staining of the infected tissues on day 3. Left scale bars are 200 μm and right scale bars are 100 μm. **i** Immunostaining of infected skin tissues stained with CD68 and DAPI on day 7. Scale bars are 100 μm. **j** Quantifications of macrophages (CD68) in the infected skin tissues. The data are presented as mean ± SD (n = 5). Statistical significance: *P < 0.05, **P < 0.01; ***P < 0.001 and ****P < 0.0001

We chose a methicillin-resistant *Staphylococcus aureus* (MRSA)-infected skin wound model in rats to explore the in vivo antibacterial and wound healing ability of the nanomaterials (Fig. 7a). After 24 h of infection, wounds were treated (day 0) with 100 µg mL^−1^ nanomaterials under 15 min irradiation. These parameters were chosen based on in vitro antibacterial and toxicity testing. Compared to the control group, the nanomaterials accelerated the wound healing process, and HTGZ showed optimal ability owing to the release of Zn^2+^ (Fig. 7b, c). Additionally, as shown by gram-positive bacteria staining and CFU counting, the number of residual bacteria from the wound sites was significantly decreased (Fig. 7d–f). Bacterial infections can seriously affect wound healing, while the excellent antibacterial activities of nanomaterials can effectively solve this issue via PDA in the initial stage.

After eliminating infection, adequate inflammation control is another barrier in wound healing. The inflammatory status of wounds is important for exploring the degree of wound healing. Inflammatory cytokine levels serve as indicators of inflammatory progression [47]. After 72 h of treatment, the serum level of interleukin (IL)-6, IL-1ß and tumor necrosis factor-α (TNF-a) in the control group was significantly higher than that in the treatment groups (Fig. 7g). To further evaluate the inflammatory conditions, histopathological and immunofluorescence techniques were employed. Hematoxylin and eosin (H&E) staining on day 3 showed fewer neutrophils emerged from the treated wounds (Fig. 7h). Furthermore, on day 7, weaker signal for cluster of differentiation 68 was observed in the treated groups relative to the control group, indicated fewer macrophages (Fig. 7i, j). During the initial stages of wound healing, neutrophils migrate from circulating blood to the wound bed, reducing microbial levels [48], while macrophages play a role in eliminating necrotic and apoptotic cells [49]. The reduced numbers of neutrophils and macrophages at the wound site suggest that treatment with nanomaterials effectively diminishes infection and inflammation during the early and middle stages of wound recovery through PDA, thereby promoting wound healing.

**Fig. 8:**
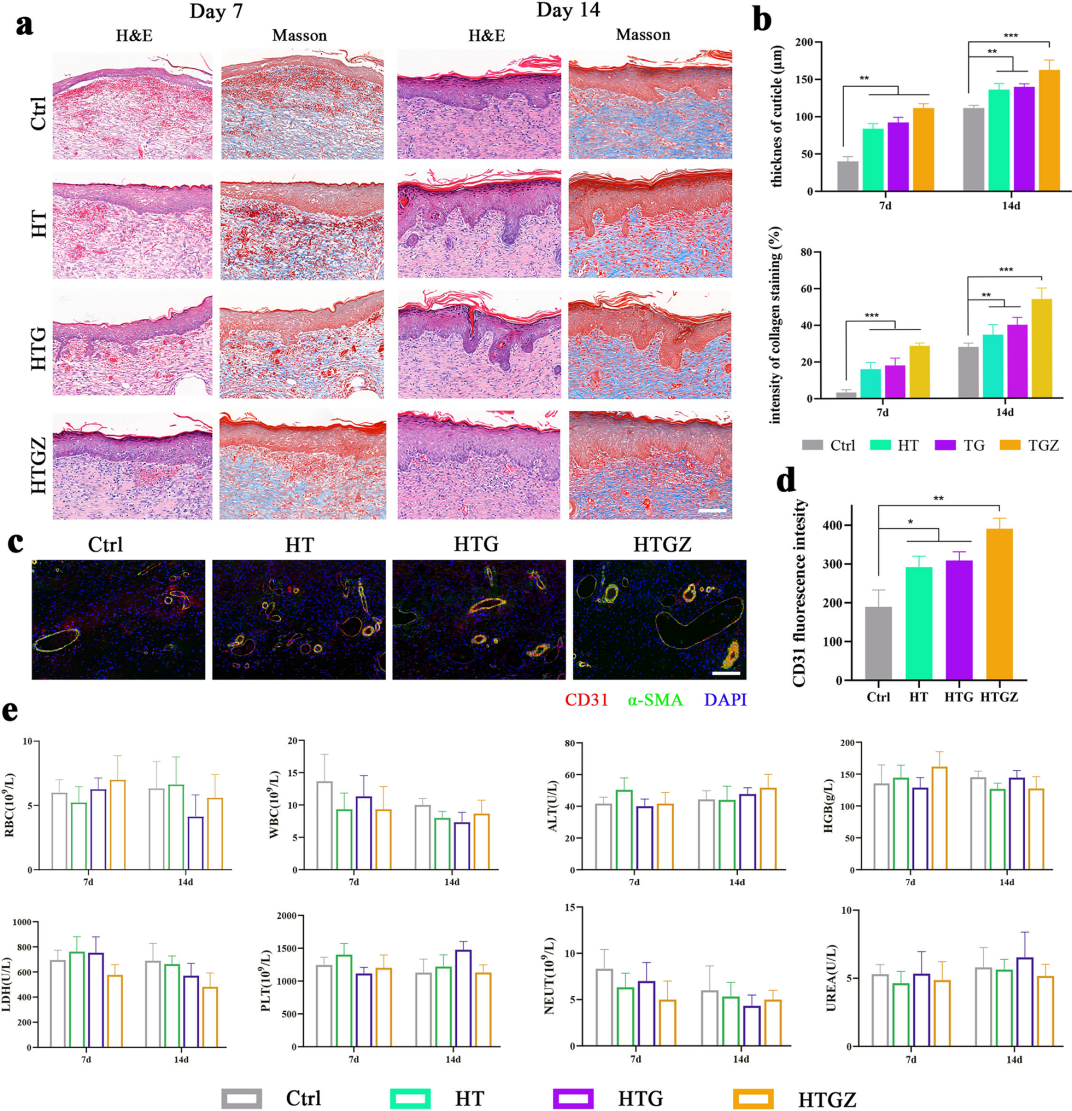
Antibacterial activity of nanomaterials in the infected wound model and periodontal inflammation model in vivo. **a** H&E and Masson staining of the infected tissues on days 7 and 14. Scale bars are 100 μm. **b** Statistical analysis of cuticle thickness and intensity of collagen staining. **c** Immunostaining of infected skin tissues stained with CD31, α-SMA and DAPI on Day 7. Scale bars are 100 μm. **d** Quantifications of CD31 in the infected skin tissues. **e** The blood routine examination and major blood biochemical values of the control and treated groups in rats. The data are presented as mean ± SD (n = 5). Statistical significance: *P < 0.05, **P < 0.01; ***P < 0.001 and ****P < 0.0001

Furthermore, we evaluated the effects of the nanomaterials on tissue regeneration using H&E and Masson’s trichrome staining. On day 7, the epidermis and dermis were not tightly bonded in the control group, whereas collagen deposition was significantly lower than that in the treated groups. On day 14, well-organized collagen fibers and the formation of skin appendages, such as follicles, were observed in the treatment groups (Fig. 8a). The quantitative analysis of epidermal thickness and collagen staining intensity are shown in Fig. 8b. Subsequently, we performed immunofluorescence staining to explore angiogenesis. CD31 is a marker for newly-formed endothelial cells [50]. while α-smooth muscle actin (α-SMA) is a marker of myofibroblasts in vascular smooth muscle [51]. The upregulation fluorescence intensity in the treated groups indicated enhanced angiogenesis (Fig. 8c, d). A thicker epidermis and increased collagen deposition indicated that the tissue was undergoing superior repair, which was indirectly confirmed by the presence of local vessels which transport the oxygen and nutrients necessary for wound healing [52].

In general, bacterial infections and subsequent inflammation seriously hinder wound repair [53]. The rapid and effective PDA properties of nanomaterials can eliminate detrimental factors that hinder wound healing by killing bacteria and alleviating inflammation. Moreover, the release of Zn^2+^ in HTGZ promotes tissue regeneration and further accelerates the overall healing process.

Additionally, the cytotoxicity of the nanomaterials was evaluated in vivo through hematological examination and histological analysis of major organs. Histological analysis using H&E staining revealed no discernible histological abnormalities in the major organs across all experimental groups (Additional file 1: Fig. S9). On day 7, the control group exhibited higher white blood cell and neutrophil counts compared to the treated groups, attributable to the presence of severe inflammation; however, all other hematological parameters remained within the normal range (Fig. 8e). These findings provide compelling evidence of the outstanding biosafety and promising application potential of nanomaterials in vivo.

Due to its enormous potential in treating infected wounds and good biosafety, HTGZ-related drugs have great practical application prospects. Because of its excellent hydrophilicity, HTGZ can be added to antibacterial patches or gels, or even directly made into aerosolized formulations.

### Alleviating periodontal inflammation in vivo

Periodontitis is a chronic inflammation characterized by loss of periodontal tissue. Plaque biofilm is an important pathogenic factor for periodontitis [54]. Therefore, the periodontitis model could further verify the antibacterial and tissue repair capacity of nanomaterials. Following different treatments, the CFU count of residual bacterial in periodontal tissue showed a significant reduction in treatment groups (Additional file 1: Fig. S10a, b). Additionally, as shown in three-dimensional reconstructed micro-CT images, compared with the control group, bone resorption area was significantly smaller in the treated groups (Additional file 1: Fig. S10c). Quantitative analysis of distance between the cemento-enamel junction (CEJ) and alveolar bone crest (ABC) at furcation of first molar confirmed this result (Additional file 1: Fig. S10d). Corresponding two-dimensional cross-sectional images revealed considerably higher bone density in the treatment groups compared to that in the control group within the region below the apex of the bone septum, between the mesial and distal roots of the first molar. The bone volume/total volume (BV/TV) within this region displayed similar trends (Additional file 1: Fig. S10e). Histopathological analysis using H&E staining revealed severe periodontal ligament width experienced and bone loss at the molar furcation area of the control group, whereas these symptoms were notably alleviated in the treatment groups (Additional file 1: Fig. S10f, g). Many positive osteoclasts (yellow arrows) were evident in the tartrate-resistant acid phosphatase (TRAP) staining image of the control group, whereas they were scarcely visible in the treatment groups (Additional file 1: Fig. S10h), suggesting the suppression of bone destruction in periodontitis. Collectively, HTGZ could eliminate biofilm through PDA, and further absorption of alveolar bone was inhibited after the initiating factors of periodontitis were removed. The symptoms of periodontitis were relieved, and periodontal tissue was regenerated.

## Conclusion

In this study, we synthesized a highly hydrophilic and dispersed TiO_2_-based nano-system HTGZ with enhanced PDA effects under visible light. HTGZ demonstrated remarkable antibacterial efficacy, achieving over 98% bacterial reduction against both *S. mutans* and *E. coli* following a mild 15 min visible light irradiation in vivo with benign cytocompatibility. The possible antibacterial mechanism of disrupting cell membranes and various metabolic processes of bacteria were also proposed. Additionally, in vitro assays demonstrated that the release of Zn^2+^ could up-regulate the expression of collagen deposition genes of L929 cells, which endowed HTGZ with considerable tissue repair capabilities. Furthermore, HTGZ exhibited satisfactory bacterial inactivation properties and wound-healing abilities in both soft and hard tissue infection in vitro. In summary, our findings provide compelling evidence that HTGZ can speed the healing of bacteria-infected impaired wound tissues, holding great promise as a therapeutic approach for the treatment of infectious bacterial diseases.

### Supplementary Information

** Additional file 1.** Materials and methods. **Figure S1.** EDS-mapping of HT, HTG and HTGZ. **Figure S2.** EDS and element composition of HTGZ. **Figure S3.** XPS spectra of nanomaterials. **Figure S4.** Antibacterial rate of nanomaterials in different concentration and under irradiation for different time. **Figure S5.** The ATP level and protein leakage of bacteria after treatment. **Figure S6.** The interaction force profile between nanomaterials and bacterial cells. **Figure S7.** The PCA, OPLS-DA plot scores and widely-untargeted metabolomics analysis. **Figure S8.** CCK-8 results of BMSCs. **Figure S9.** H& E staining of major organs. **Figure S10.** Antibacterial activity of nanomaterials in periodontal inflammation model in vivo.

## Data Availability

The data that support the findings of this study are available from the corresponding author upon reasonable request.
