# Supplementary material for: Highly hydrophilic and dispersed TiO2 nano-system with enhanced photocatalytic antibacterial activities and accelerated tissue regeneration under visible light
Source: J Nanobiotechnology. 2023 Dec 20;21:491. doi: 10.1186/s12951-023-02241-2 (PMC10731761; doi:10.1186/s12951-023-02241-2)
Supplement: Supplementary file 1 — Additional file 1. Materials and methods. Figure S1. EDS-mapping of HT, HTG and HTGZ. Figure S2. EDS and element composition of HTGZ. Figure S3. XPS spectra of nanomaterials. Figure S4. Antibacterial rate of nanomaterials in different concentration and under irradiation for different time. Figure S5. The ATP level and protein leakage of bacteria after treatment. Figure S6. The interaction force profile between nanomaterials and bacterial cells. Figure S7. The PCA, OPLS-DA plot scores and widely-untargeted metabolomics analysis. Figure S8. CCK-8 results of BMSCs. Figure S9. H& E staining of major organs. Figure S10. Antibacterial activity of nanomaterials in periodontal inflammation model in vivo. [file 12951_2023_2241_MOESM1_ESM.docx]

Additional file 1

**Highly Hydrophilic and** **Dispersed TiO_2_ Nano-system with Enhanced Photocatalytic Antibacterial Activities and Accelerated Tissue Regeneration Under Visible Light**

*Boyao Lu^#1^, Jie Zhang^#2^, Guixin Zhu^1^, Tiqian Liu^1^, Jinwei Chen*^2,3^, Xing Liang*^1^*

^1^ State Key Laboratory of Oral Diseases & National Center for Stomatology & National Clinical Research Center for Oral Diseases, Department of ProsthodonticsⅡof West China Hospital of Stomatology, Sichuan University, Chengdu 610041, Sichuan, China

^2^ College of Materials Science and Engineering, Sichuan University, Chengdu 610065, China

^3^ Engineering Research Center of Alternative Energy Materials & Devices, Ministry of Education, Sichuan University, Chengdu 610065, China

^*^ Corresponding authors:

Jinwei Chen: jwchen@scu.edu.cn; and Xing Liang: liangxing@scu.edu.cn

^#^ Boyao Lu and Jie Zhang contributed equally to this work.

**MATERIALS AND METHODS**

**Synthesis of Materials**

HT was prepared using a facile, non-aqueous sol–gel method. Briefly, 5 mL TiCl_4_ was slowly dropped to 40 mL anhydrous ethanol under ambient conditions with strong magnetic stirring. The sol was transferred to a stainless-steel autoclave and stored at 180 °C for 3 h. The resulting powder was washed with deionized water and vacuum-dried overnight. HTG was prepared in the same way with 5 mL of GO (0.56 mg mL^-1^ in ethanol); ZnCl_2_ (0.24 g) was also added to prepare the HTGZ.

**Materials Characterizations**

The morphology and microstructure of the nanomaterials were investigated by scanning electron microscope (SEM, JSM-6510LV, JEOL, Japan) and transmission electron microscopy (TEM, FEI Talos, US). The energy-dispersive spectroscopy (EDS) and element distribution mapping was conducted combined with SEM. The physical analysis of phase structure was verified by X-ray diffraction (XRD, D8A25, Bruker, Germany). The zeta potential was performed by dynamic light scattering analysis (Nano ZS90, Malvern Instruments Ltd., Malvern, UK). The molecular structure was detected by Raman spectroscopy (Renishaw Co. RM1000). The functional groups change were determined by Fourier transform infrared spectroscopy (FTIR, Nicolet 6700, Thermo fisher, US). The elemental analysis was examined using X-ray photoelectron spectroscopy (XPS; AXIS Ultra DLD, Kratos, UK). The UV–vis diffuse reflectance spectrum (DRS) was performed on UV3600 (Shimadzu, Japan). The surface contact angles were detected by a contact angle meter (Powereach® JC2000C1).

**Optical Characterizations and Photodynamic Effect**

The ability of nanomaterials to generate ·OH and ·O2^-^ was measured using electron spin resonance (ESR, Bruke, EMX-Plus) at room temperature. The measurement was conducted in dark and repeated after 30 minutes irradiation. The electrochemical impedance spectroscopy (EIS) and photocurrent measurements were used an electrochemical analyzer (CHI 760B, Shanghai Chenhua Instruments Ltd, China). The ROS generation of nanomaterials under visible light was measured using a Reactive Oxygen Species Assay Kit (Beyotime, China). The fluorescence intensity was measured on a spectrophotometric microplate reader before and after light irradiation.

**Rhodamine B (RhB) Degradation Assay**

100 mL of 10 mg mL^-1^ RhB solution and 10 mg of photocatalysts were added. After standing for 10 minutes to achieve adsorption equilibrium, the solution was exposed to visible light (420 nm < λ < 780 nm) carried out by an Xenon lamp at a power of 300 mWcm^−2^ for 60 min. The solution (5 mL) was removed and centrifuged at 10,000 g for 10 min at certain time intervals. Absorbance of the supernatant was measured using a UV spectrophotometer (SOPTOP, UV2800, China).

**Bacterial Culture**

*Streptococcus mutans,* (*S. mutans*, UA159, ATCC) were cultured in brain heart infusion (BHI) medium and *Escherichia coli* (*E. coli*, 25922, ATCC) were cultured in Luria-Bertani (LB) medium. These two bacteria were cultured under 37°C. The density of bacteria suspensions was adjusted to 1 × 10^9^ colony-forming unit (CFU) mL^-1^ for further use.

***In Vitro* Anti Planktonic bacteria Assays**

Nanomaterials at different concentrations and bacterial solutions were added to 24-well plates. The irradiation groups were irradiated with visible light at 300 mWcm^−2^. At fixed time intervals (5, 10, and 15 min), 100 μL of the bacterial suspension in each group was collected and serially diluted in phosphate-buffer solution (PBS). The bacterial diluent (100 µL) was plated onto agar plates and cultured at 37 °C for 24 h. The relative bacterial viability was estimated by the following Equation (1):

Relative bacterial viability (%) = CFU _sample_ / CFU _control_= ×100% (1)

where “CFU _sample_” and “CFU _control_” are the number of colonies of treatment groups and control group (bacteria without nanomaterials and irradiation at 0). In addition, samples treated with 200 µg mL^-1^ nanomaterials for 15min were then incubated at 37 °C for 18 h. Optical density (OD) values were recorded every hour at a wavelength of 600 nm using a microplate reader and the bacterial growth curve was plotted. All assays were performed three times.

**Bacteria Morphology Observation**

The bacteria morphology was examined by SEM and TEM. For SEM, bacteria were fixed with 2.5% glutaraldehyde overnight and dehydrated in a sequential ethanol series. Then, the samples were dried and coated with gold for observation. For TEM, bacteria were prefixed with 2.5% glutaraldehyde for 4h and postfixed in 1% osmium tetroxide for 2 h and dehydrated in series acetone and embedded in Epox812. Thereafter, resin pellets were cut with diamond knife into ultrathin sections and stained with uranyl acetate and lead citrate for observation.

**Bacteria Live/Dead Staining**

Bacteria treated with 200 µg mL^-1^ nanomaterials for 15min were stained with SYTO 9/ PI (Thermo Fisher L13152). After washing with PBS, bacterial samples were imaged using laser scanning confocal microscopy (LSCM, Fv3000; Olympus, Japan).

**Adenosine Triphosphate (ATP) Test**

The ATP level was evaluated using an Enhanced ATP Assay Kit (Beyotime, China). Bacteria treated with 200 µg mL^-1^ nanomaterials for 15 min were centrifuged at 10,000 g for 5 min at 4 °C and suspended in lysis buffer. The samples were analyzed according to the manufacturer’s instructions.

**Protein Leakage Test**

The protein leakage level was evaluated using an Enhanced BCA Protein Assay Kit (Beyotime, China). Bacteria treated with 200 µg mL^-1^ nanomaterials for 15 min were centrifuged at 10,000 g for 5 min at 4 °C. The upper liquid was collected and analyzed according to the manufacturer’s instructions.

***In Vitro* anti-biofilm Assays**

*S. mutans* treated with 200 µg mL^-1^ nanomaterials for 15min were added into 24-well plates and intubated in BHI- sucrose medium 1 d to establish biofilms. Crystal violet method was used to measured the ability of nanomaterials to inhibit biofilm formation. 200 µL crystal violet solution (1% w/v) was added to each well and stained for 30 min. The biofilm was visualized using a stereomicroscope (Leica, Germany). Thereafter, 95% ethanol solution was added and100 µL of each solution was monitored using a microplate reader at 570 nm. In addition, fluorescently labeled live/dead assay and CFU counts were used to detected the observe viability of bacteria in the treated mature biofilms. *S. mutans* biofilm was established according to previous protocols and then treated with 200 µg mL^-1^ nanomaterials for 15min. Live/dead staining was performed and observed as described above. Meanwhile, the adherent biofilm was scraped off and resuspended in PBS by vigorous pipetting and vortexing. 100 μL of the bacterial suspension in each group was collected and diluted. The bacterial diluent (100 µL) was plated onto agar plates and cultured as planktonic bacteria.

**Interaction Force Measurement**

After immersed in 0.1% (w/v) poly-L-lysine for 1 min, the cantilever of tipless AFM probes (HQ: CSC38/tipless/AlBS, Ultrasharp, μ-Masch, Tallinn, Estonia) was immersed in the bacteria suspension for 2 min to adhere cells to the surface. One drop of nanomaterials suspension (200 μg mL^–1^) was placed on a clean mica sheet. After air drying, the interaction force was examined under contact model by atomic force microscopy (AFM, Shimadzu SPM-9700, Japan).

**Intracellular ROS Detection**

The intracellular ROS levels of bacteria were estimated by 2′,7′-dichlorofluorescein-diacetate (DCFH-DA; Beyotime, China). Bacteria treated with 200 µg mL^-1^ nanomaterials for 15min. Afterwards, the samples were incubated with fresh medium containing ROS probe at 37 °C for 60 min. After rinsing with PBS, the fluorescence images were captured using LSCM.

**Transcriptome Analysis and Metabolome Analysis**

*E. coli* were co-cultured with 50 µg mL^-1^ HTGZ for 10 min under visible light illumination in HTGZ treated (TR) group. Bacteria in control (CL) group didn’t receive any treatment. Then the bacteria cells were washed by deionized water and collected by centrifugation. The cell pellets were placed into liquid nitrogen for 15 min immediately and stored at −80 °C after freezing. Total RNA extraction, RNA sequencing, metabolite extraction and bioinformatic data collection were performed by Personalbio (Nanjing, China).

**Cell Culture**

L929 cells were cultured in Dulbecco’s modified Eagle’s medium (DMEM; Gibco, US) and rat bone mesenchymal stem cells (BMSCs) were cultured in Alpha-minimum Eagle’s medium (a-MEM; Gibco, US). All the medium contained 10% fetal bovine serum (FBS; Gibco, US) and 1% penicillin- streptomycin (Gibco, US). The cells were maintained at 37 °C in humidified incubators with 5% CO_2_ supply.

**Cell Viability Assay**

The effect of the nanomaterials on cell viability and proliferation was evaluated by Cell Counting Kit-8 (CCK-8) test. The cells were co-cultured with the nanomaterials for 1, 2, or 4 days. After adding the CCK-8 solution, the samples were incubated for 90 min, and the optical density (OD) was measured at 450 nm by a spectrophotometric microplate reader. The relative cell viability was estimated by the following Equation (2):

Relative bacterial viability (%)=OD _sample_ / OD _control_= ×100% (2)

where “OD _sample_ ”and “OD _control_” are the OD _450_ values of the well for treatment groups and control group (cells without nanomaterials at 1day).

**Cell Morphology Observation**

The cells were co-cultured with 100 µg mL^-1^ nanomaterials for 1 day. Afterwards, the samples were fixed with 4% paraformaldehyde, permeabilized with 0.5% Triton X-100 and cleaned with PBS. Last, the samples were stained with FITC-Phalloidin (YiSen, Shanghai) for 30 min and DAPI (YiSen, Shanghai) for 60 s in darkness. The images were captured using LSCM.

**Cells Live/dead Staining**

The cells were co-cultured with 100 µg mL^-1^ nanomaterials for 1 day. Afterwards, the samples were washed with PBS and stained by Calcein/PI Cell Viability/Cytotoxicity Assay Kit (Beyotime, China). The images were captured using LSCM.

**Hemolysis Assay**

The blood collected from Sprague–Dwaley (SD) rat aorta abdominal was prepared to 2% (w/v) erythrocyte suspension. 100 µg mL^-1^ nanomaterials were added to experimental group, respectively, in positive and negative control group, deionized water and saline was added. The samples were incubated at 37 °C for 4 h. Afterwards, the OD of upper liquid was measured at 540 nm by a spectrophotometric microplate reader.

**Cell Scratch Experiment**

1 × 10^5^ L929 cells were cultured in each well of 24-well plates to 95% coverage. Then, a scratch of uniform width was made by a sterile 1 mL pipet tip and the shed cells were washed and removed by PBS. The culture medium with 50 μg mL^-1^ of nanoparticles was added to the well. The growth and migration of cells were recorded in bright-field at 0, 18, and 36 h using inverted optical microscopy.

**Quantitative Real Time Reverse Transcription Chain Reaction (qRT-PCR)**

The L929 cells were co-cultured with nanomaterials of 50 µg mL^-1^ for 2, 4 or 6 days. The total RNA was extracted according to manufacturer’s instructions using RNAsimple Total RNA Kit (TIANGEN) and reversely transcribed to cDNA with PrimeScript™ RT reagent kit (Takara). qRT-PCR was performed using 384-well Optical Reaction Plate (Axygen) and iTaq Universal SYBR Green Supermix (Biorad). The primers sequences for each targeted genes are listed in Table 1.

***In Vivo* Infected Wound Healing Test and Biosafety Assessment**

60 Male Sprague Dawley (SD rats; 6-week-old) were purchased from Sichuan University. All animal experiments were approved by the Animal Ethics Committee of Sichuan University. After one week of acclimatization, a circular wound 10 mm in diameter was made on the back. The MRSA suspension (50 µL, 10^8^ CFU mL^−1^) was added to each wound area to establish the infected wound model for 24 h. The rats were randomly divided into four groups with 15 rats in each group and treated with 50µL nanomaterials (100 µg mL−1) or PBS. The wounds were irradiated with visible light for 15 min (day 0). Bacteria in the infected wound area were collected and numbered on day 1. Blood from randomly selected rats in each group was collected on day 3 to measure serum levels of inflammatory cytokines using an ELISA kit (ZCIBIO, China) Tissue sections of infected samples were collected and stained with hematoxylin and eosin (H&E) and Gram stain on day 3; H&E and Masson’s trichrome on day 7 and 14. Rats were sacrificed in each group on day 7 for cluster of differentiation 68 (CD68), CD31 and α-smooth muscle action (α-SMA) immunofluorescent staining. Blood samples were obtained for hematological examination on days 7 and 14, and the major organs (heart, liver, spleen, lung, and kidney) were harvested and subjected to H&E staining on day 14. Photographs of the wounds were obtained on days 0, 3, 7, 10 and 14.

***In Vivo* Periodontitis Healing Model**

20 Male C57BL/6 mice (8-week-old) were purchased from Sichuan University. After one week of acclimatization, A 6–0 silk suture was ligated around the cervical region of the maxillary first molar to create experimental periodontitis model. After two weeks, the rats were randomly divided into four groups with 5 rats in each group. 50 µL nanomaterials (100 µg mL−1) or PBS were administered for oral rinsing and irradiated under visible light for 15 min once every other day for one week. Afterwards, the animals were sacrificed and the maxillaries were surgically dissected and collected for further evaluation. The bacteria adhered on the teeth surface were collected and numbered. The microarchitecture of the teeth was scanned using a micro-CT (mCT-50, Scanco Medical Inc, Switzerland). Simultaneously, the distance between cemento-enamel junction (CEJ) to alveolar bone crest (ABC) and the bone volume/total volume (BV/TV) were measured. Then, the maxillary tissues with teeth were decalcified for 4 weeks and embedded in paraffin for H&E and tartrate-resistant acid phosphatase (TRAP) staining.

**Statistical Analysis**

The results of the experiments are presented as mean ± standard deviation (SD) The data among each group were analyzed by One way analysis of variance (ANOVA) combined with Tukey’s post-test to assess statistic difference. P values < 0.05 was considered to be statistically significant (*P < 0.05, **P < 0.01, ***P < 0.001, and ****P < 0.0001).

**Table 1.** qRT-PCR primers.

| Gene | Forward primer sequence (5’–3’) | Reverse primer sequence (5’–3’) |
| --- | --- | --- |
| MMP-2 | F-5'-CTTCCAAGTCTGGAGCGATGT-3' | R-5‘-TACCGTCAAAGGGGTATCCAT-3' |
| COL-I | F-5'-TGCTGATGGACAACCTGG-3' | R-5'-ACTGTTGCCTTTGGGACC-3' |
| COL-III | F-5'-CTGGATCTCCTGGTGCTAAG-3' | R-5'-CAGCGTGTCCTTGTGGTC-3' |

**ADDITIONAL FIGURES**


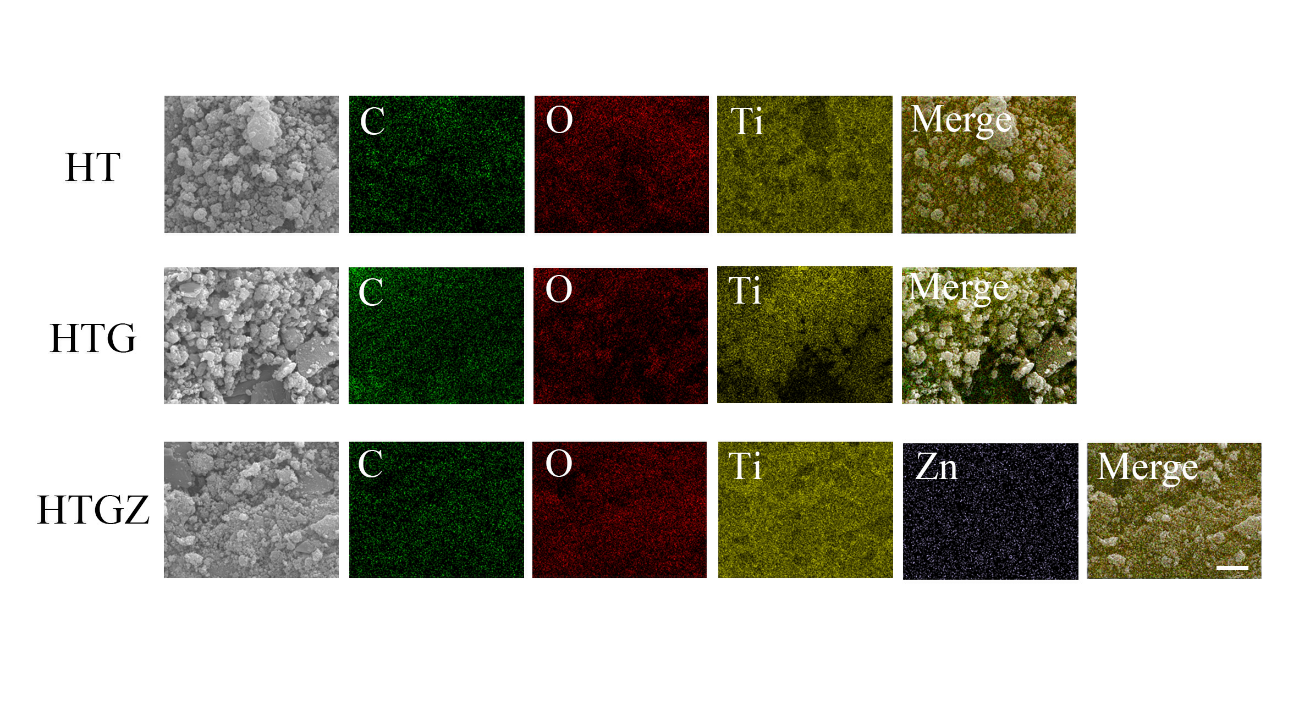


**Figure S1.** EDS-mapping of HT, HTG and HTGZ. Scale bars are 2 μm.


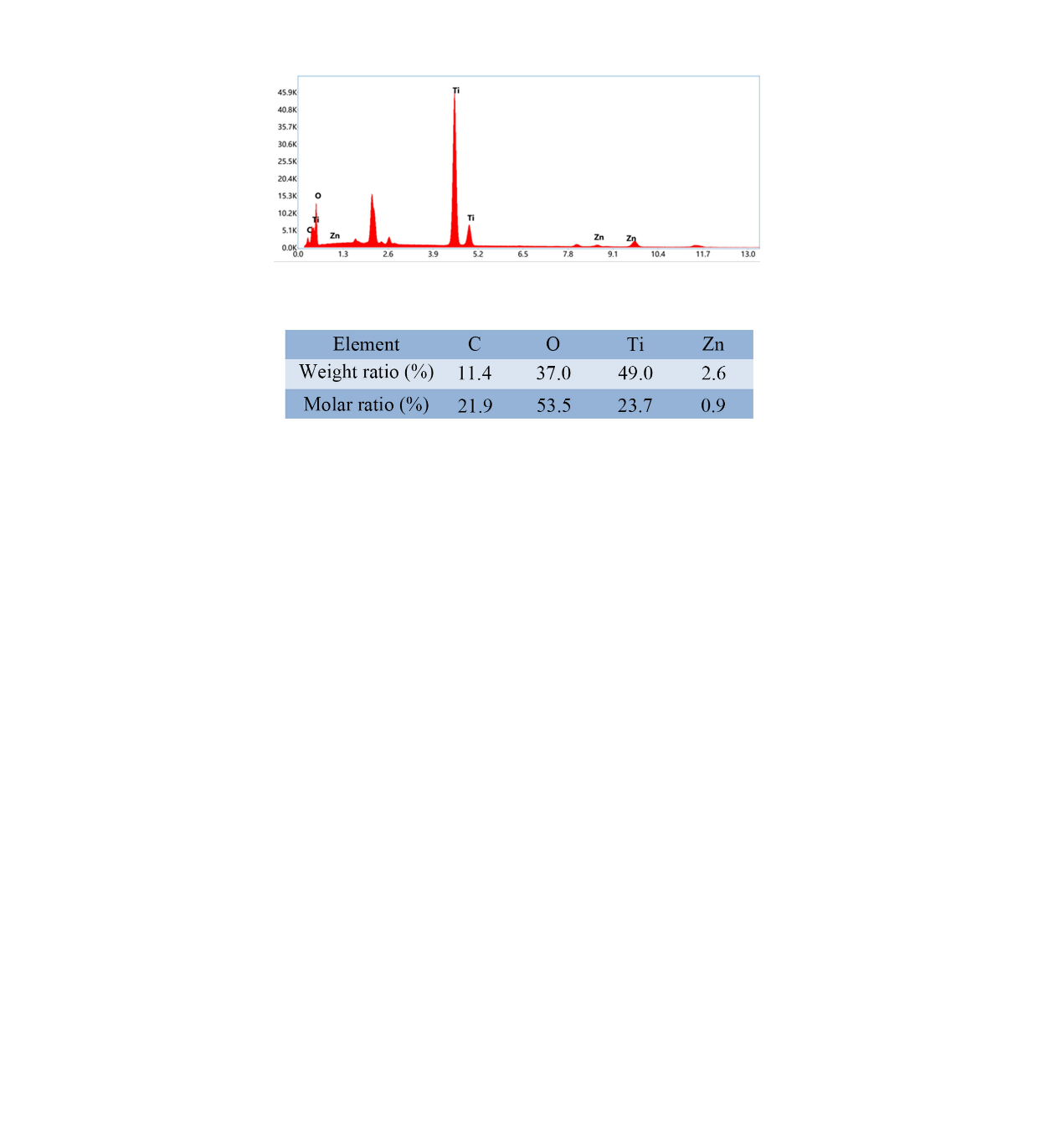


**Figure S2.** EDS spectrum and the element composition of HTGZ.


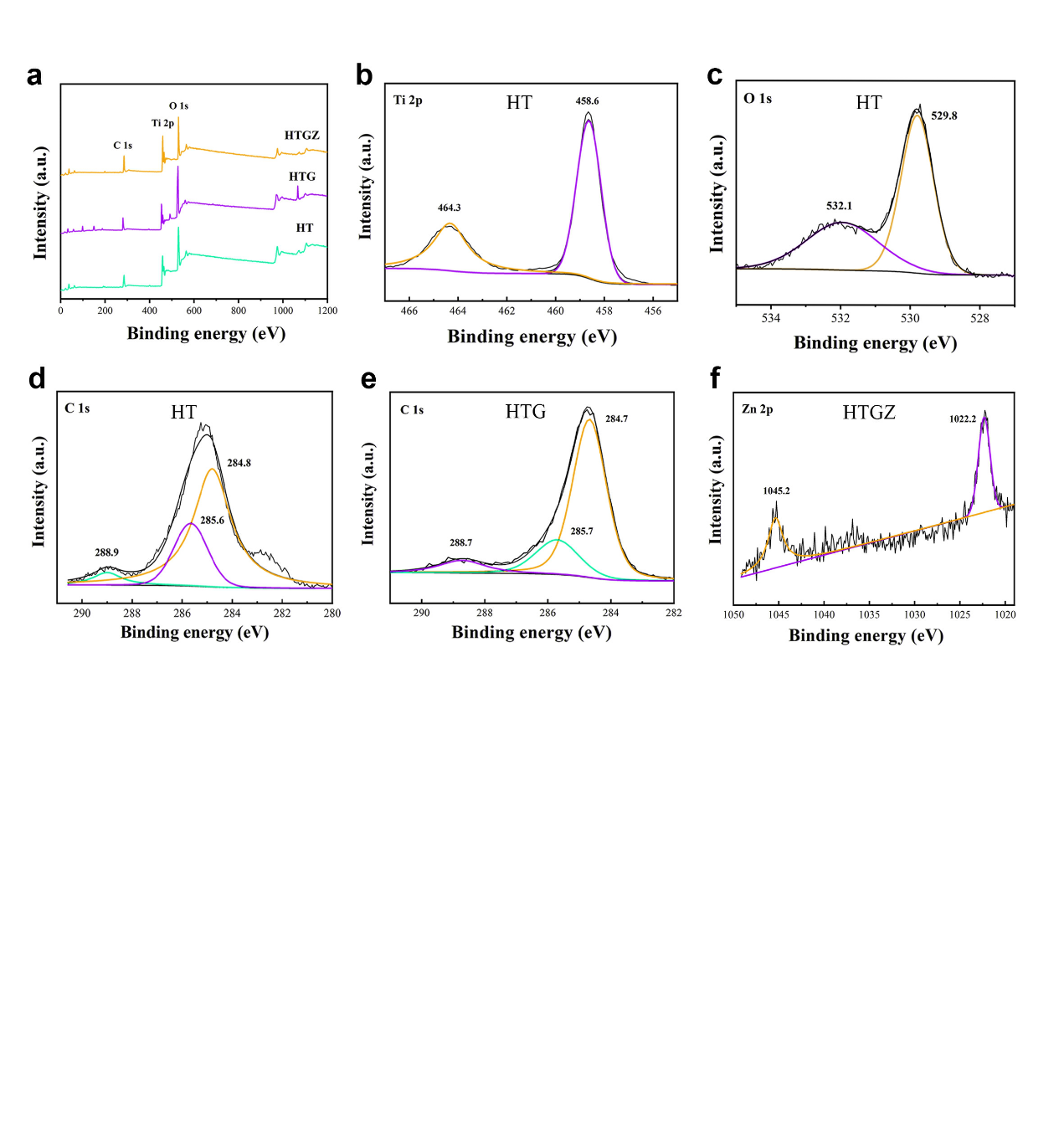


**Figure S3.** XPS spectra of nanomaterials. (a) XPS survey spectra of HT, HTG and HTGZ. High-resolution XPS spectra of (b) Ti 2p in HT, (c) O 1s in HT, (d) C 1s in HT, (e) C 1s in HTG, and (f) Zn 2p in HTGZ.


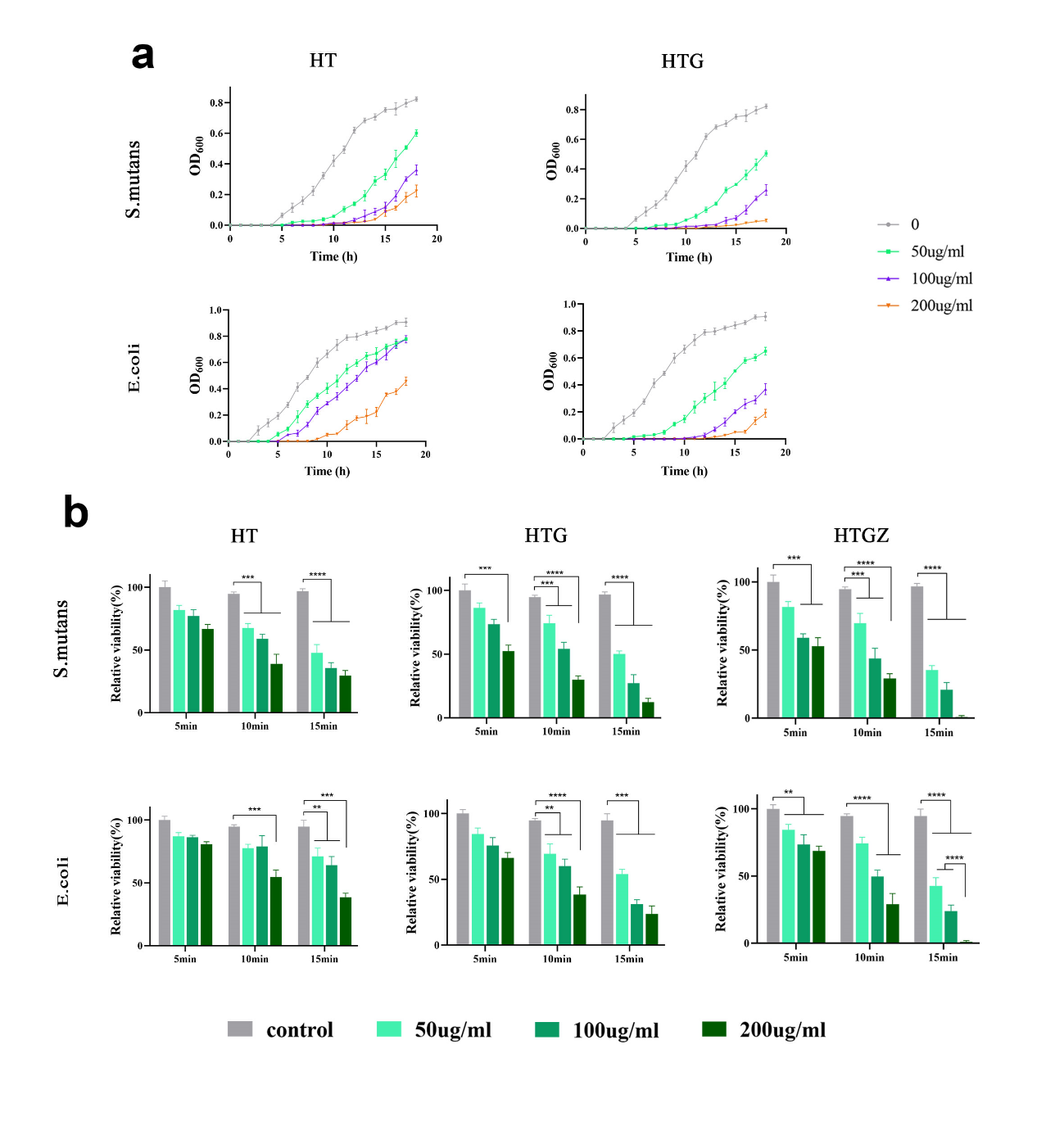


**Figure S4.** Antibacterial effects of nanomaterials in different concentration and under irradiation for different time. (a) The growth curve of bacteria. (b) The loss of viability of bacteria. The data are presented as mean ± SD (n = 3). Statistical significance: *P < 0.05, **P < 0.01, ***P < 0.001 and ****P < 0.0001.


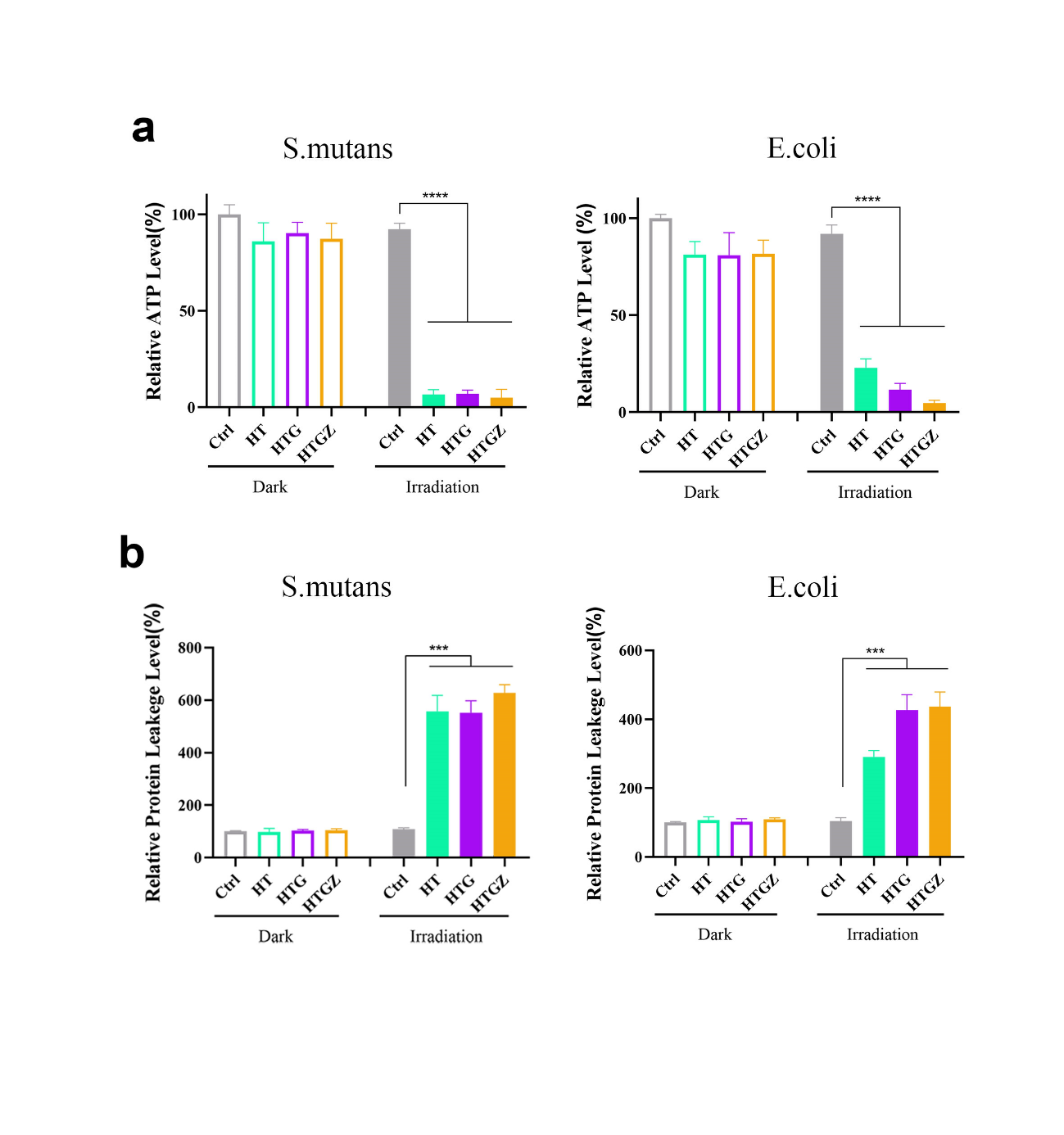


**Figure S5.** (a) The ATP level of bacteria after treatment with 200 µg mL^−1^ nanomaterials for 15 min. (b) Protein leakage of bacteria after treatment with 200 µg mL^−1^ nanomaterials for 15 min. The data are presented as mean ± SD (n = 3). Statistical significance: *P < 0.05; **P < 0.01; ***P < 0.001 and ****P < 0.0001.


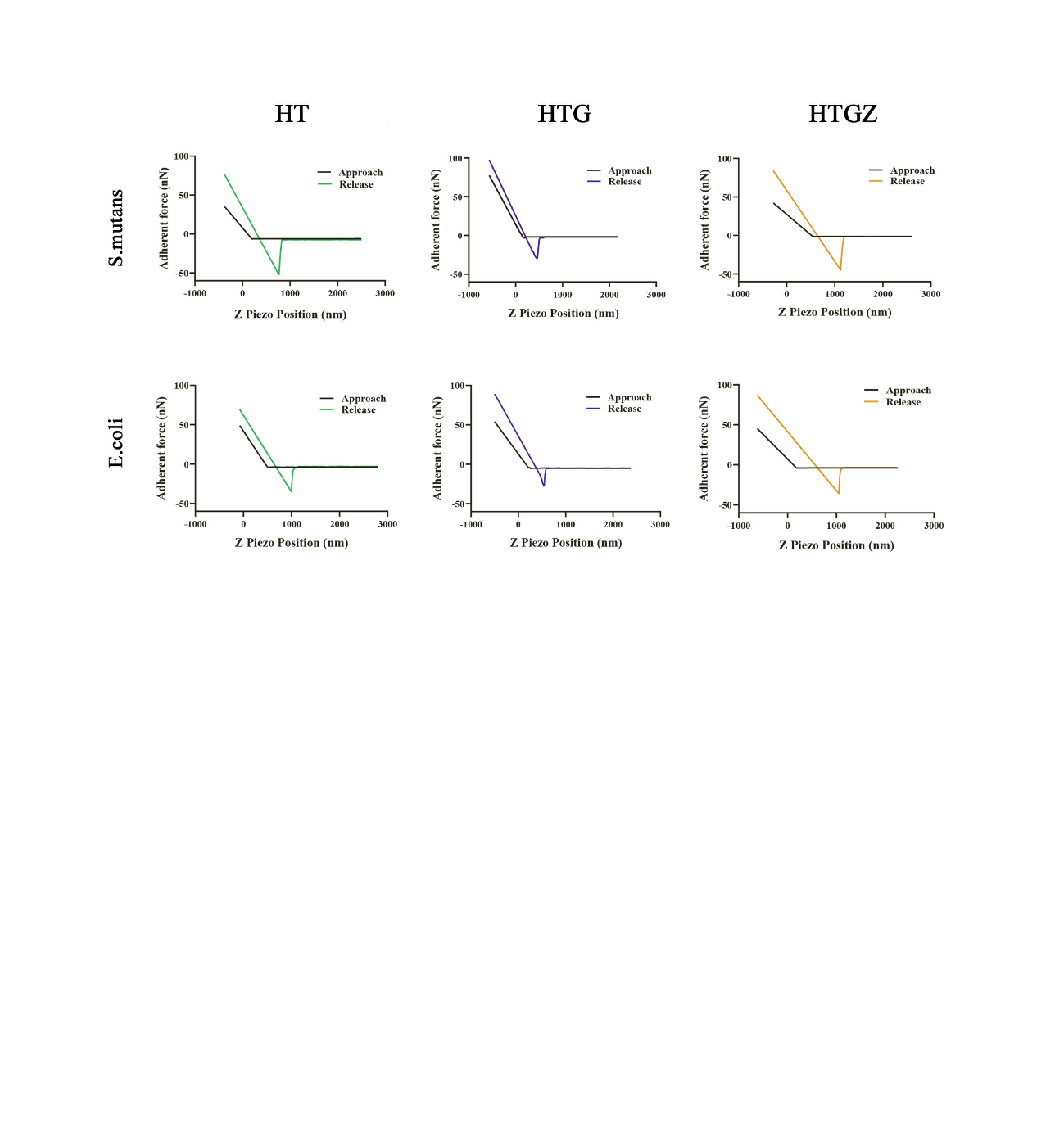


**Figure S6.** The representative interaction force profile between nanomaterials and bacteria cells examined by AFM.


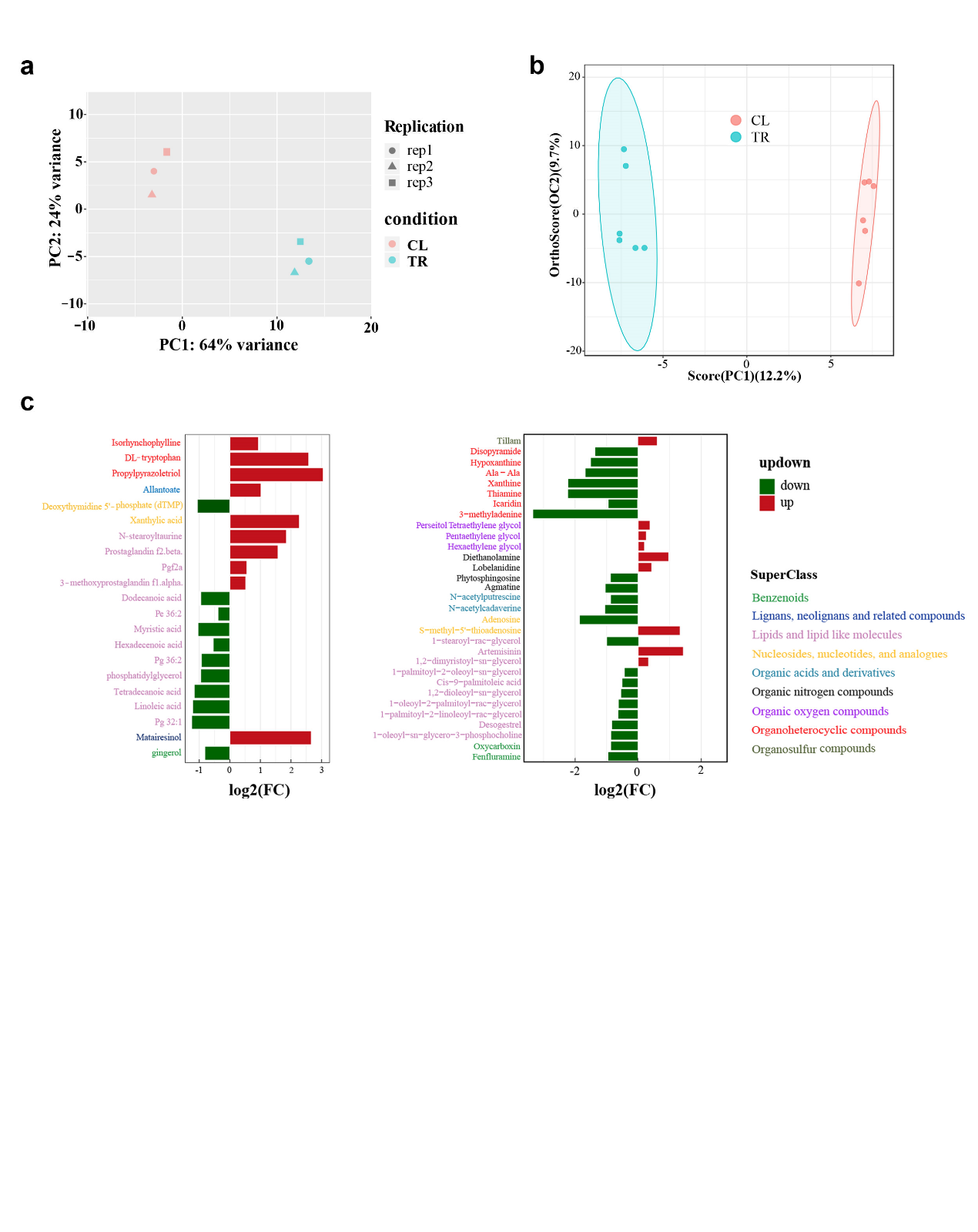


**Figure S7.** (a) The PCA plot scores were derived from the transcriptomics of the CL and TR groups. (b) The OPLS-DA plot scores were derived from the metabolic profiling of the CL and TR groups. (c) Widely untargeted metabolomics analysis revealed the DEMs and their log2Foldchange in CL versus TR with positive and negative ion mode.


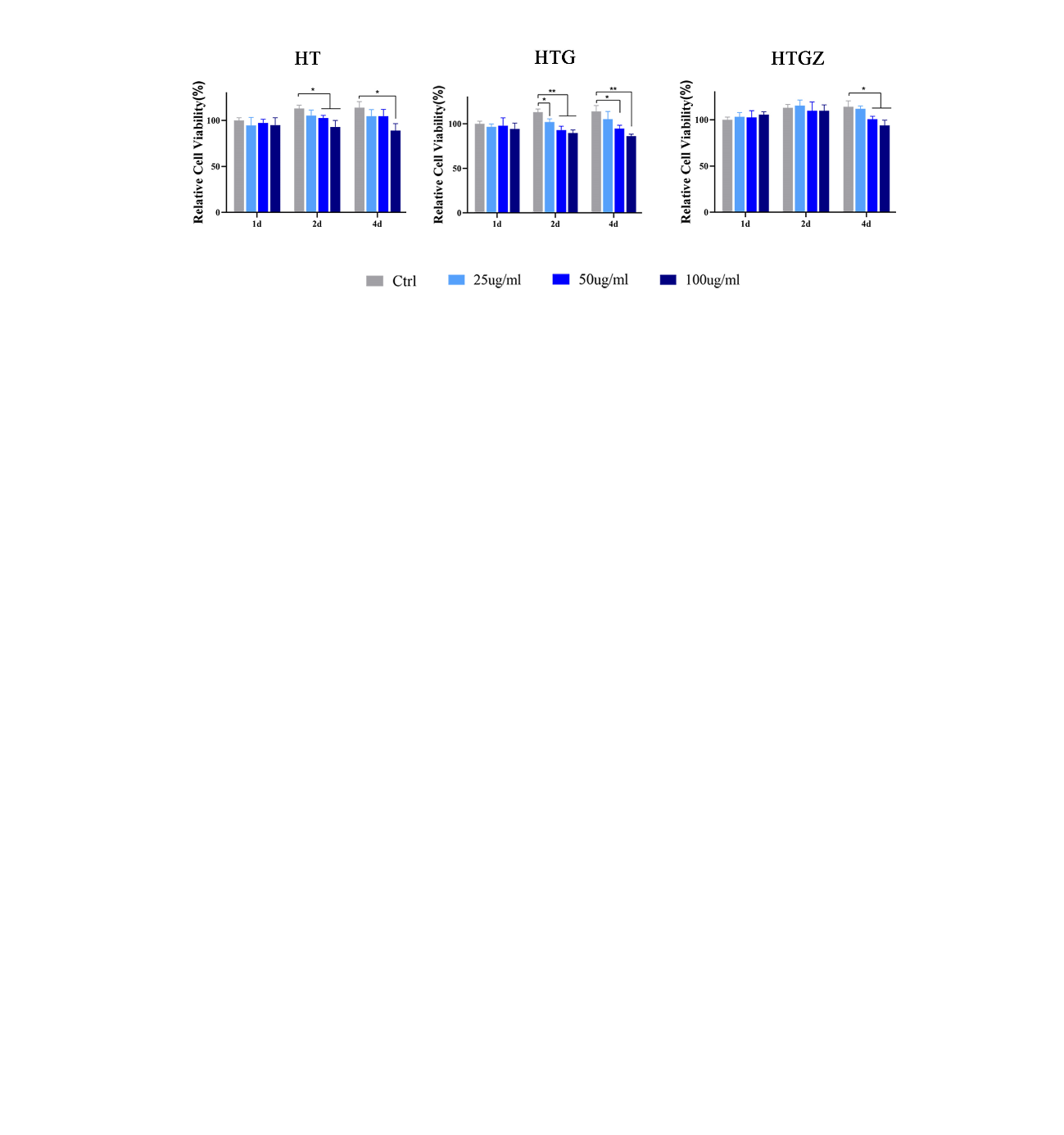


**Figure S8.** CCK-8 results of BMSCs co-cultured with nanomaterials.

**
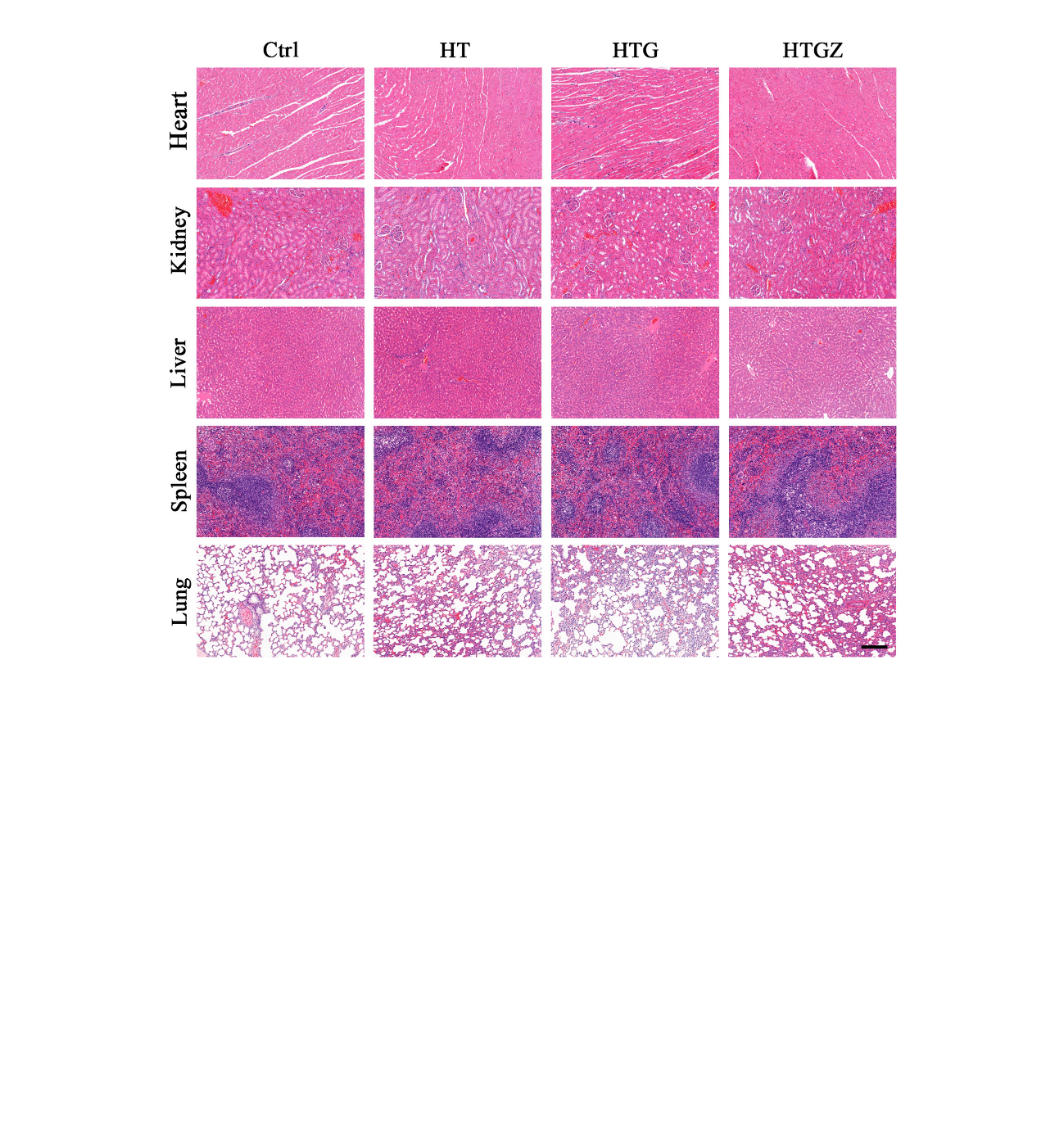
**

**Figure S9.** H&E staining of major organs (heart, liver, spleen, lung, and kidney) in days 14. Scale bars are 200 μm.


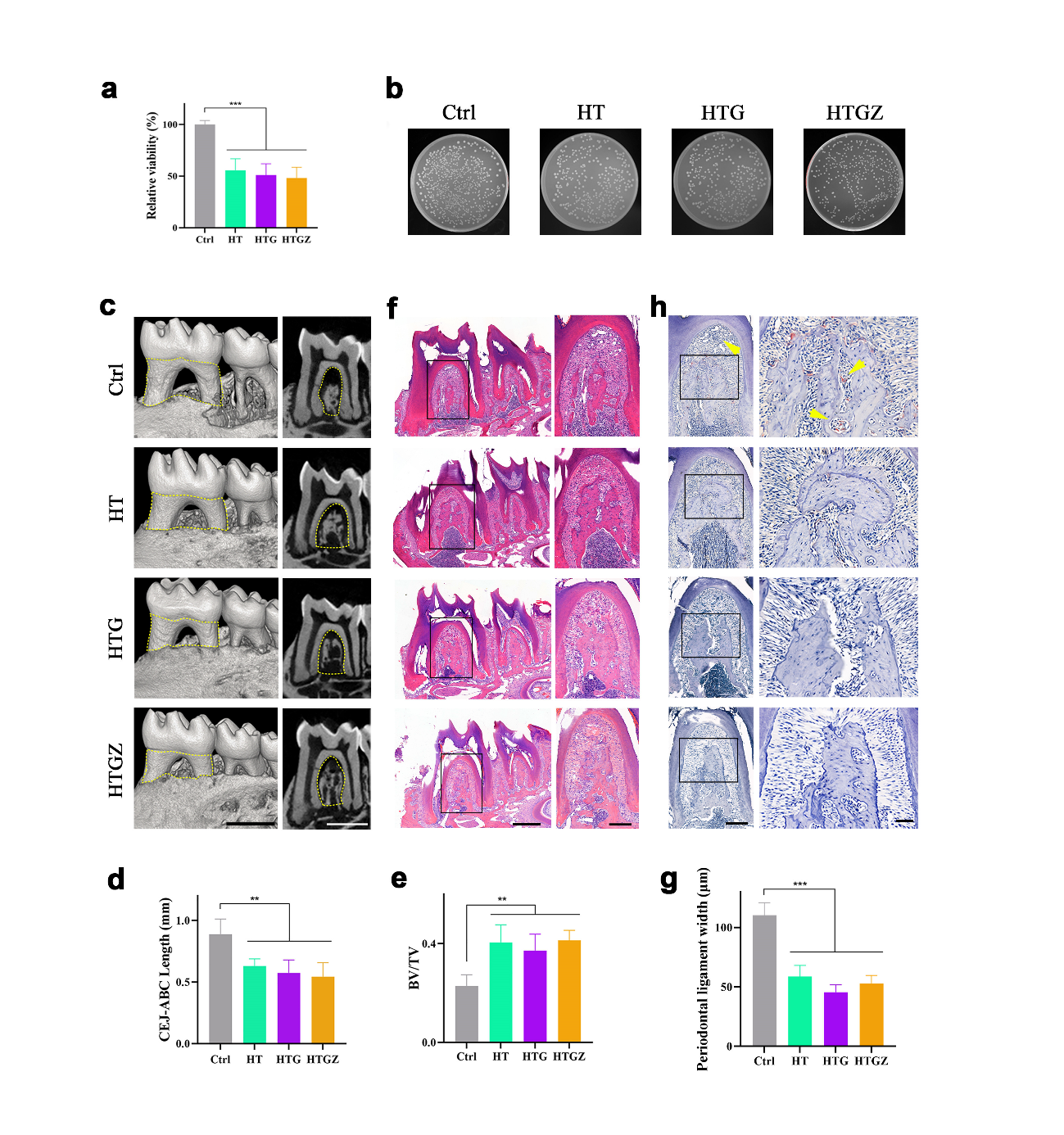


**Figure S10.** Antibacterial activity of nanomaterials in periodontal inflammation model *in vivo*. (a) Quantitative analysis of CFUs from the surface of the mice tooth. (b) Photographs of bacterial colonies. (c) Representative 3D reconstructed sections (left) and their corresponding micro-CT scanning images (right). Bone resorption region and the area for calculating bone volume was indicated by the yellow-dotted. Scale bars are 1 mm. (d) Quantification of the distance from CEJ to ABC in different groups. (e) Quantification of BV/TV measured for alveolar bone areas in different groups. (f) Histologic changes in periodontal tissue were monitored by hematoxylin and eosin (H&E) staining. Left scale bars are 500 µm and right scale bars are 200 µm. (g) Quantitative analysis of the periodontal ligament width in different groups. (h) Representative images of TRAP-stained in different groups. The yellow arrow indicated TRAP-positive osteoclasts. Left scale bars are 100 µm and right scale bars are 50 µm. The data are presented as mean ± SD (n = 5). Statistical significance: *P < 0.05, **P < 0.01; ***P < 0.001 and ****P < 0.0001.
